# *cdc-25.4*, a *Caenorhabditis elegans* Ortholog of *cdc25*, Is Required for Male Mating Behavior

**DOI:** 10.1534/g3.116.036129

**Published:** 2016-10-21

**Authors:** Sangmi Oh, Ichiro Kawasaki, Jae-Hyung Park, Yhong-Hee Shim

**Affiliations:** Department of Bioscience and Biotechnology, Konkuk University, Seoul 05029, Republic of Korea

**Keywords:** CDC-25.4 phosphatase, male mating, turning behavior, neuronal expression, *Caenorhabditis elegans*

## Abstract

Cell division cycle 25 (cdc25) is an evolutionarily conserved phosphatase that promotes cell cycle progression. Among the four *cdc25* orthologs in *Caenorhabditis elegans*, we found that *cdc-25.4* mutant males failed to produce outcrossed progeny. This was not caused by defects in sperm development, but by defects in male mating behavior. The *cdc-25.4* mutant males showed various defects during male mating, including contact response, backing, turning, and vulva location. Aberrant turning behavior was the most prominent defect in the *cdc-25.4* mutant males. We also found that *cdc-25.4* is expressed in many neuronal cells throughout development. The turning defect in *cdc-25.4* mutant males was recovered by *cdc-25.4* transgenic expression in neuronal cells, suggesting that *cdc-25.4* functions in neurons for male mating. However, the neuronal morphology of *cdc-25.4* mutant males appeared to be normal, as examined with several neuronal markers. Also, RNAi depletion of *wee-1.3*, a *C. elegans* ortholog of Wee1/Myt1 kinase, failed to suppress the mating defects of *cdc-25.4* mutant males. These findings suggest that, for successful male mating, *cdc-25.4* does not target cell cycles that are required for neuronal differentiation and development. Rather, *cdc-25.4* likely regulates noncanonical substrates in neuronal cells.

Cdc25 phosphatase promotes cell cycle progression by removing an inhibitory phosphate from cyclin-dependent kinase 1 (Cdk1), which is placed by Wee1/Myt1 kinase (Donzelli and Draetta 2003) . Cdc25 is a highly conserved phosphatase that is present from yeast to mammals (Edgar and O’Farrell 1989; Jimenez *et al.* 1990; Sadhu *et al.* 1990; Forsburg and Nurse 1991). In *C. elegans*, four *cdc-25* genes, *cdc-25.1*, *cdc-25.2*, *cdc-25.3*, and *cdc-25.4*, have been identified (Ashcroft *et al.* 1998). *cdc-25.1* and *cdc-25.2* are required for germline mitotic proliferation (Ashcroft *et al.* 1999; Ashcroft and Golden 2002; Kim *et al.* 2009; Yoon *et al.* 2012) and for oocyte meiotic maturation (Kim *et al.* 2010), respectively. The roles of *cdc-25* genes are not confined to the germ cells. *cdc-25.1* acts at the G1/S transition in the embryonic E lineage, and stabilization of CDC-25.1 in the E lineage in *cdc-25.1*(*gf*) mutants causes an increase in the number of intestinal cells (Hebeisen and Roy 2008; Segref *et al.* 2010). *cdc-25.2* is also required for proper progression of characteristic intestinal divisions (Lee *et al.* 2016). These results demonstrate that *cdc-25* genes are involved in both germline and somatic development. However, the functions of *cdc-25.4* remain largely unknown.

Intriguingly, the functions of these cell cycle regulators are not limited to control of the cell cycle. A variety of cell cycle regulators are now known to be involved in other biological processes, including regulation of transcription, epigenetics, metabolism, and neuronal functions (Lim and Kaldis 2013). For example, CDK10 suppresses the transcriptional activity of the transcription factor ETS2 via a direct interaction in human cells (Kasten and Giordano 2001). CDK1 and CDK2 phosphorylate enhancer of zeste homolog 2, the catalytic subunit of Polycomb repressive complex 2, in human prostate cancer cells, and this phosphorylation regulates the methyltransferase activity of the complex, which leads to transcriptional repression of target genes (Chen *et al.* 2010). The functions of CDK5 in neuronal development in multiple organisms have been reported (Su and Tsai 2011). In *C. elegans*, CDK-5 regulates polarized trafficking of presynaptic components and dense-core vesicles (Ou *et al.* 2010; Goodwin *et al.* 2012). CDK-5 is also required for synapse elimination and formation (Park *et al.* 2011), and for trafficking of glutamate receptors in the ventral nerve cord (Monteiro *et al.* 2012). CDK-8 is required for axon navigation decisions in neurons (Steimel *et al.* 2013), and also functions in vulval development by regulating the epidermal growth factor receptor-Ras-extracellular signal-regulated kinase pathway (Grants *et al.* 2016). Taken together, these results suggest that CDKs phosphorylate not only cell cycle regulators, but also other regulators that are involved in diverse, important biological processes.

Male mating behavior, which is regulated by multiple neuronal networks, is one of the most complex behaviors observed in *C. elegans*. *C. elegans* contains a limited number of neuronal cells. Hermaphrodites have 302 neurons out of 959 somatic cells, and males have 385 neurons out of 1031 somatic cells (Sulston and Horvitz 1977). Among them, 170 neurons, which include 81 male-specific neurons and 89 neurons common to both sexes, function in male mating processes, in collaboration with 64 muscles (Jarrell *et al.* 2012). Notably, most of these cells are located in the male tail region (Sulston *et al.* 1980). The male mating process can be divided into several stages as follows: (1) recognition of a hermaphrodite by touching her body with his tail, (2) moving backward until reaching either the head or tail of her body, (3) turning, (4) location of his tail at the vulva, (5) spicule insertion, and (6) ejaculation (Loer and Kenyon 1993; Liu and Sternberg 1995). All of these steps are closely associated with each other and integrated together in the nervous system via male-specific neurons including rays, CP, hook, and postcloacal sensilla neurons (Barr and Garcia 2006).

Here, we report, for the first time, that *cdc-25.4* regulates male mating behavior. We found that *cdc-25.4* is expressed in many neurons throughout development. Depletion of *cdc-25.4* activity resulted in male sterility caused by defects in male mating behavior. The turning defect was the most prominent defective phenotype displayed by *cdc-25.4* mutant males. This defect was rescued by *cdc-25.4* transgene expression driven by the endogenous *cdc-25.4* promoter, as well as by several neuronal promoters, indicating that *cdc-25.4* activity is required in neurons for successful male mating. Furthermore, *cdc-25.4* mutant males showed several additional defects, such as contact response, backing, and vulva location defects, suggesting that *cdc-25.4* is required in multiple processes during mating. Interestingly, the turning defect of *cdc-25.4* mutant males was not suppressed by depletion of *wee-1.3*, unlike canonical cell cycle regulations by CDC-25.1 and CDC-25.2, such as those in germline mitotic proliferation, oocyte maturation, and intestinal divisions, in which defects of *cdc-25* mutants were largely suppressed by depletion of *wee-1.3*, a *C. elegans* ortholog of Wee1/Myt1 kinase (Kim *et al.* 2010; Yoon *et al.* 2012; Lee *et al.* 2016). This result suggests that *cdc-25.4* does not function in the regulation of the cell cycle in the male nervous system. Rather, *cdc-25.4* may have a unique noncanonical function in neuronal cells that is vital for successful male mating behavior.

## Materials and Methods

### Strains and maintenance

*C. elegans* strains were grown as described previously (Brenner 1974). The Bristol N2 strain or *him-5*(*e1467*) was used as wild-type controls for all experiments. The mutant alleles used in this study were as follows: LGI; *ttTi4348*; LGII; *cdc-25.4*(*tm4088*), *cat-2*(*e1112*); LGIII; *glp-1*(*q231*), *unc-119*(*ed3*), *bas-1*(*ad446*); LGIV; *fem-1*(*hc17*), *fem-3*(*q20*), *unc-22*(*e66*); and LGV; *fog-2*(*q71*), *him-5*(*e1467*). The following transgenic reporters were used in this study: *egIs1[dat-1p*::*GFP]*, *kkuIs05[Pcdc-25.4*::*gfp*::*cdc-25.4*::*cdc-25.4 3′ UTR*; *cb-unc-119*(*+*)*] I*, *kkuEx06[Pcdc-25.4*::*gfp*::*cdc-25.4 3′ UTR*; *Prab-3*::*mCherry*::*unc-54 3′ UTR*; *cb-unc-119*(*+*)*]*, *kkuEx08[Ptph-1*::*gfp*::*cdc-25.4*::*unc-54 3′ UTR*; *Pmyo-2*::*mCherry*::*unc-54 3′ UTR]*, *kkuEx09[Punc-119*::*gfp*::*cdc-25.4*::*unc-54 3′ UTR*; *Pmyo-2*::*mCherry*::*unc-54 3′ UTR]*, *kkuEx15[Ptba-9*::*gfp*::*cdc-25.4*::*unc-54 3′ UTR*; *Pmyo-2*::*mCherry*::*unc-54 3′ UTR]*, *kkuEx16[Ppkd-2*::*gfp*::*cdc-25.4*::*unc-54 3′ UTR*; *Pmyo-2*::*mCherry*::*unc-54 3′ UTR]*, *mgIs42[tph-1p*::*GFP + pRF4*(*rol-6*(*su1006*))*]*, *otIs45[unc-119*::*GFP] V*, *oxEx1580[eft-3p*::*GFP*; *cb-unc-119*(*+*)*]*, and *uIs60[unc-119p*::*YFP + unc-119p*::*sid-1]*. All strains were maintained at either 15 or 20° on nematode growth medium (NGM) agar plates containing *Escherichia coli* strain OP50. Males were generated either by introducing the *him-5*(*e1467*) mutation to the strain or by heat shock (Hodgkin 1983).

### Quantitative real-time RT-PCR

Total RNA was prepared from wild-type N2 hermaphrodite populations synchronized at each of four (L1–L4) larval stages and at the adult (A) stage, from an N2 adult male population, and from *fem-1*(*hc17lf*), *fem-3*(*q20gf*), and *glp-1*(*q231*) adult hermaphrodites grown at 25°. Total RNA and cDNA were prepared and qPCR reactions were performed as previously described (Kim *et al.* 2010). The primers for *act-1*, which served as the internal control, were 5′-CCA GGA ATT GCT GAT CGT ATG CAG AA-3′ and 5′-TGG AGA GGG AAG CGA GGA TAG A-3′. The primers for *cdc-25.4* were 5′-GAC AGG TAT CAG ACT AGA TTC TC-3′ and 5′-CAG CAC CCT TAA TAT GTC CA-3′. The relative expression level of each gene was defined as the mRNA level of each gene averaged from triplicate experiments and then normalized to that of *act-1*.

### Male fertility test

To test *cdc-25.4*(*tm4088*) male fertility, we used *fog-2*(*q71*) mutants. *Fog-2*(*q71*) hermaphrodites are sterile owing to an absence of sperm (Schedl and Kimble 1988). For the fertility test, fresh NGM plates seeded with a 150 µl spot of OP50 overnight culture were prepared. A single L4 larval *fog-2*(*q71*) hermaphrodite and a single mutant male were placed on an OP50-spotted NGM plate and allowed to mate at 20°. They were transferred to a new OP50-spotted NGM plate every day for 3 d and the total number of outcrossed progeny was counted. As the negative and positive controls, single *fog-2*(*q71*) hermaphrodite-alone plates and single *fog-2*(*q71*) hermaphrodite mated with single *him-5*(*e1467*) male plates, respectively, were included in each experiment.

### Immunofluorescence analysis

To observe the meiotic division processes during spermatogenesis, immunofluorescence analysis was performed as previously described (Kim *et al.* 2010). Briefly, adult virgin males were dissected and extruded gonads were fixed with cold methanol and 3% paraformaldehyde. The fixed specimens were incubated with 1:200-diluted FITC-conjugated anti-α-tubulin (mouse monoclonal, Sigma) and 1:200-diluted anti-phospho-Histone H3 (rabbit polyclonal, Upstate) as primary antibodies at 4° overnight. Alexa 546-conjugated donkey anti-rabbit IgG (1:500 dilution) (Molecular Probes) was used as the secondary antibody. The specimens were counterstained with 0.5 μg/ml Hoechst 33342 to visualize DNA.

### In vitro sperm activation

*In vitro* sperm activation was carried out as previously described (Singaravelu *et al.* 2011) with some minor modifications. L4 males were isolated on OP50-seeded NGM plates and cultured on the plates at 20° for 2 d in the absence of hermaphrodites. Then, 10 of the virgin males were transferred to 7 μl of 1 × sperm medium (50 mM HEPES, 25 mM KCl, 45 mM NaCl, 1 mM MgSO_4_, 5 mM CaCl_2_, and 10 mM Dextrose; pH 7.8) with or without 2 mg/ml of pronase on a glass slide. Spermatids were released by cutting the tails. After incubating at RT for 5 min, a coverslip was gently overlaid and sealed with vaseline. Activation of spermatids to spermatozoa was observed at 630 × magnification under Nomarski differential interference contrast (DIC) microscopy.

### Sperm transfer assay

To examine the transfer of sperm from a male to a hermaphrodite during mating, a single L4 male was mated with a single L4 *fog-2*(*q71*) hermaphrodite on a fresh OP50-spotted NGM plate at 20° for 2 d. After mating, the *fog-2*(*q71*) hermaphrodites were dissected, and extruded gonads were fixed with cold methanol and cold acetone. Then, the fixed gonads were immunostained with a sperm-specific mouse monoclonal antibody SP56 (a gift from Susan Strome), as previously described (Kim *et al.* 2010), to detect successfully transferred sperm in the gonads. Briefly, the fixed gonads were first incubated with 1:100-diluted SP56 at 4° overnight, followed by incubation with 1:500-diluted Alexa 488-conjugated goat anti-mouse IgG (Molecular Probes) secondary antibody at room temperature (RT) for 3 hr. DNA was counterstained with 0.5 µg/ml Hoechst 33342. For DNA staining of male gonads, virgin adult males were dissected, and extruded gonads were fixed with cold methanol and 3% paraformaldehyde. Then, the DNA in the fixed gonads was stained with 0.5 µg/ml Hoechst 33342.

### Mating behavior assay

Observation of mating behavior was carried out as previously described (Liu *et al.* 2007). Before the assay, L4 males were isolated on an OP50-seeded NGM plate and incubated at 20° overnight. NGM plates were prepared 1 d prior to the experiments, and an OP50 overnight culture 3 mm in diameter was spotted on the plates. When the culture had been completely absorbed into the plates (after several hours), the OP50-spotted NGM plates were used for the following mating behavior assay: a single virgin 1-d-old adult male was placed on a mating plate that contained twenty evenly-distributed immotile *unc-22*(*e66*) adult hermaphrodites. Mating behavior was observed for 15 min or until ejaculation. Animals maintained at 20° were either observed at 65 × magnification under a Nikon SMZ1500 microscope or recorded at 63 × magnification using a Hamamatsu Orca ERG digital camera under a Zeiss Axioskop 2 MOT microscope with Nikon NIS-Elements Basic Research imaging software (version 4.3). Mating behavior was classified as previously described (Loer and Kenyon 1993; Koo *et al.* 2011). Percent contact response = (the number of successful contact responses/total number of male tail contacts with a hermaphrodite) × 100. Both tail contact and the initiation of backing are required for a successful contact response. Percent backing = (the number of completed backward locomotion/total number of backing attempts) × 100. Completed backward locomotion was defined as a male moving backward until reaching either the head or tail of a hermaphrodite. Turning behavior was classified and scored as previously described (Loer and Kenyon 1993). Completed turns consist of both good and sloppy turns. Percent vulva location and spicule insertion were also calculated by dividing the number of successful behaviors with total number of trials. Response time was measured from the time when a male was placed on a mating plate to the time of his first successful contact response.

### Male tail-chasing behavior

Ten young adult males were transferred to a fresh NGM plate seeded with 10 µl of OP50. Male tail-chasing behavior was observed for 30 min at 20 × magnification. When a male circled at least once and touched his head to his tail, tail-chasing behavior was counted as one success.

### Construction of transgenic reporter DNAs

All plasmid DNAs were constructed using the In-Fusion HD Cloning system (Clontech). A 6.8 kb genomic sequence spanning from 4.9 kb upstream from the *cdc-25.4* start codon to 0.9 kb downstream from the *cdc-25.4* stop codon was PCR-amplified from N2 genomic DNA using primer pairs specifically designed for the In-Fusion reaction. The amplified genomic sequence was ligated by an In-Fusion reaction with pCFJ352 vector DNA, which was linearized by double restriction enzyme digestion with *Spe*I and *Nco*I. Finally, a *gfp* coding sequence with artificial introns, which was PCR-amplified from the pJA256 vector, was inserted into the N-terminal end of the *cdc-25.4* coding sequence to generate the *Pcdc-25.4*::*gfp*::*cdc-25.4*::*cdc-25.4* 3′ UTR translational reporter construct. The *Pcdc-25.4*::*gfp* transcriptional reporter construct was generated by removing the *cdc-25.4* coding sequence from the translational reporter construct through inverse PCR and In-Fusion. *Pmyo-3*::*mCherry*::*unc-54* 3′ UTR (pCFJ104) was used to generate neuronal promoter-driven *cdc-25.4* rescue constructs. First, pCFJ104 DNA was linearized by inverse PCR using primers designed at the end of *Pmyo-3* and the beginning of *unc-43* 3′ UTR sequences. Then, a *gfp*::*cdc-25.4* fusion sequence, which was PCR-amplified from the *Pcdc-25.4*::*gfp*::*cdc-25.4*::*cdc-25.4* 3′ UTR translational reporter construct, was inserted into the linearized pCFJ104 DNA by the In-Fusion reaction. The *myo-3* promoter sequence was replaced with the promoter sequences of *unc-119* (2.3 kb) (Maduro and Pilgrim 1995), *tph-1* (3.2 kb) (Sze *et al.* 2000), *tba-9* (2 kb) (Hurd *et al.* 2010), or *pkd-2* (2 kb) (Barr and Sternberg 1999), which were PCR-amplified from N2 genomic DNA using specific primers. The vectors, pCFJ352, pJA256, pCFJ104, pGH8, and pCFJ90, were obtained from Addgene.

### Generation of transgenic animals

Transgenic animals were generated using standard microinjection techniques (Mello and Fire 1995), except that the *Pcdc-25.4*::*gfp*::*cdc-25.4*::*cdc-25.4* 3′ UTR (10 ng/µl) construct was integrated into the *ttTi4348* locus on chromosome I using the MosSCI technique (Frøkjær-Jensen *et al.* 2008). The DNA constructs were injected at the following concentrations: *Pcdc-25.4*::*gfp*::*cdc-25.4* 3′ UTR (20 ng/µl) with a coinjection marker, *Prab-3*::*mCherry* (10 ng/µl, pGH8); *Punc-119*::*gfp*::*cdc-25.4*::*unc-54* 3′ UTR (25 ng/µl), *Ptph-1*::*gfp*::*cdc-25.4*::*unc-54 3′* UTR (50 ng/µl), *Ptba-9*::*gfp*::*cdc-25.4*::*unc-54 3′* UTR (50 ng/µl), and *Ppkd-2:gfp*::*cdc-25.4*::*unc-54 3′* UTR (50 ng/µl) with a coinjection marker; and *Pmyo-2*::*mCherry* (2 ng/µl, pCFJ90).

### Microscopy

Transgenic worms were observed and recorded under an Olympus FV-1000 spectral confocal microscope with FV10-ASW 2.0 software. Specimens were observed and recorded under a Zeiss Axioskop 2 MOT fluorescence microscope with Openlab software (Improvision). Images were processed with Photoshop CC and Illustrator CC2015 software (Adobe).

### Neurotransmitter treatment

Treatment with neurotransmitters was performed as previously described (Loer and Kenyon 1993; Sawin *et al.* 2000; LeBoeuf *et al.* 2014) with minor modifications. 100 mM serotonin creatinine sulfate monohydrate (5-HT, Sigma) and 200 mM dopamine hydrochloride (DA, Sigma) were freshly prepared in M9 buffer. 5-HT was dissolved in solution by heating at 65°. After autoclaved NGM agar medium had cooled, 5-HT and DA were added at 1 and 2 mM final concentrations, respectively. The NGM agar plates were dried at RT for several hours and OP50 overnight cultures with 1 mM 5-HT or 2 mM DA were spotted. The plates were allowed to dry at RT overnight in the dark. Virgin young adults were transferred to the plates containing neurotransmitters and incubated at 20° for 1–2 hr (5-HT) or 6–7 hr (DA). Turning behavior was observed as described previously.

### RNA interference

RNAi was performed by the soaking method as previously described (Yoon *et al.* 2012). For PCR amplification of cDNA templates, the yk cDNA clone, yk321c10, for *wee-1.3* was generously provided by Y. Kohara (National Institute of Genetics, Japan). *cdc-25.4* cDNA was amplified from an N2 cDNA using gene-specific primers. Double-stranded (ds) RNA was transcribed *in vitro* from the PCR-amplified cDNA templates and purified as previously described (Maeda *et al.* 2001). Synchronized L1 worms were soaked in the dsRNA solution at 20° for 24 hr, allowed to recover on OP50-seeded NGM plates, and grown at 20° until they reached the adult stage.

### Statistical analysis

All the experiments were repeated more than three times for statistical evaluation of data. *P* values were calculated by Student’s *t*-test. *P* < 0.05 was considered as statistically significant.

### Data availability

Strains and reagents are available upon request. The authors state that all data necessary for confirming the conclusions presented in the article are represented fully within the article.

## Results

### cdc-25.4 is required for male fertility

**Figure 1:**
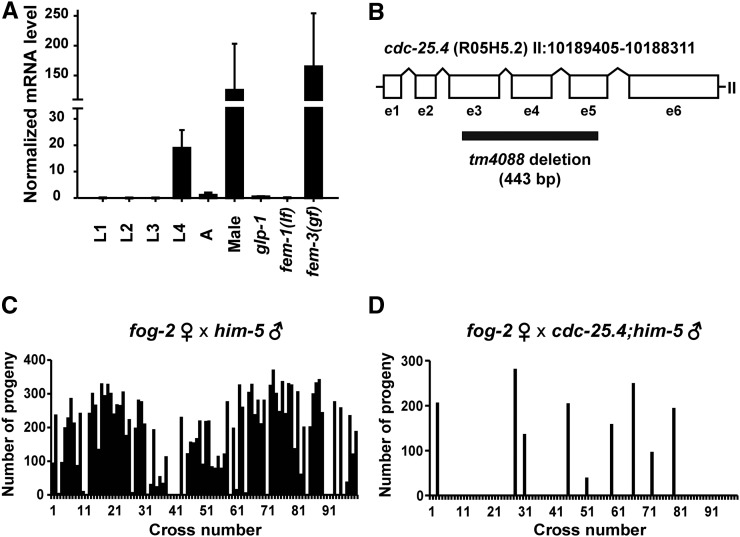
*cdc-25.4* is required for male reproduction. (A) *cdc-25.4* mRNA (messenger RNA) expression levels were examined in larval (L1–L4) and adult (A) stages as well as in males, germline-proliferation-defective *glp-1*(*q231*) mutants, *fem-1*(*hc17lf*) mutants that produce only oocytes, and *fem-3*(*q20gf*) mutants that produce only sperm. The values were normalized to those of *act-1*. Error bars indicate SD. (B) Genomic structure of *cdc-25.4* (R05H5.2), which is located on chromosome II. *cdc-25.4* consists of six exons (e1–6) and five introns. The region of the *tm4088* deletion (443 bp) is also shown. (C) The number of progeny produced by *fog-2*(*q71*) single hermaphrodites after mating with single *him-5*(*e1467*) males (*n* = 98). (D) The number of progeny produced by *fog-2*(*q71*) single hermaphrodites after mating with single *cdc-25.4*(*tm4088*); *him-5*(*e1467*) males (*n* = 97). Of *cdc-25.4*; *him-5* males, 90.7% (88/97) failed to produce outcrossed progeny.

Expression levels of *cdc-25.4* mRNA were examined at different larval stages in wild-type N2 hermaphrodites and in N2 adult males by qRT-PCR. *cdc-25.4* mRNA was highly expressed in N2 adult males, and at the L4 larval stage, when maturation of reproductive organs actively occurs, in N2 hermaphrodites (Figure 1A). We also found that *cdc-25.4* mRNA was abundantly expressed in *fem-3*(*q20gf*) hermaphrodites which produce only sperm (Barton *et al.* 1987), but not in *glp-1*(*q231*) hermaphrodites which contain few germ cells (Austin and Kimble 1987), or in *fem-1*(*hc17lf*) hermaphrodites which produce only oocytes (Nelson *et al.* 1978). These results suggest that *cdc-25.4* should play a critical role in male reproduction.

To study the roles of *cdc-25.4*, we first observed the phenotype of *cdc-25.4*(*tm4088*) deletion mutants, in which 443 bp, encompassing from exon 3 (e3) to exon 5 (e5), is deleted (Figure 1B). *cdc-25.4*(*tm4088*) mutant hermaphrodites are viable and fertile. Their brood size does not differ from that of wild-type hermaphrodites at 20° (Supplemental Material, Figure S1). To analyze the phenotype of *cdc-25.4* mutant males, we introduced the *him-5*(*e1467*) mutation, which generates a substantial number of males among self-progeny (Hodgkin 1983), into the *cdc-25.4* mutant background. First, we examined *cdc-25.4*(*tm4088*) male fertility to verify that *cdc-25.4* functions in male reproduction. To test male fertility, a single *fog-2*(*q71*) hermaphrodite was mated with either a single *him-5*(*e1467*) male or a single *cdc-25.4*(*tm4088*); *him-5*(*e1467*) male, and the numbers of outcrossed progeny were scored. In *fog-2*(*q71*) hermaphrodites, the germline is completely feminized and spermatogenesis does not occur during the L4 larval stage (Schedl and Kimble 1988). Therefore, sperm must be provided by males for *fog-2*(*q71*) hermaphrodites to produce progeny. As expected, *fog-2*(*q71*) hermaphrodites incubated alone were all sterile (*n* = 30, data not shown). When *fog-2*(*q71*) hermaphrodites were mated with *him-5*(*e1467*) males, 87.8% of the *fog-2*(*q71*) hermaphrodite parents successfully produced outcrossed progeny (*n* = 98) (Figure 1C). However, when *fog-2*(*q71*) hermaphrodites were mated with *cdc-25.4*(*tm4088*); *him-5*(*e1467*) males, 90.7% of the *fog-2*(*q71*) hermaphrodite parents failed to produce outcrossed progeny (*n* = 97) (Figure 1D). Notably, several *fog-2*(*q71*) hermaphrodite parents that successfully produced outcrossed progeny after being mated with *cdc-25.4*; *him-5* males generated similar numbers of outcrossed progeny as *fog-2*(*q71*) hermaphrodite parents that were mated with *him-5* control males. This result suggests that, although the rate of successful mating was much lower than the control *him-5* males, if mating was successful, *cdc-25.4*; *him-5* males could transfer similar numbers of functional sperm to *fog-2* hermaphrodites. Taken together, based on the results of expression analysis and male mating tests, we conclude that *cdc-25.4* is required for either male mating behavior or male fecundity.

### Sperm development is not defective in cdc-25.4 mutants

**Figure 2:**
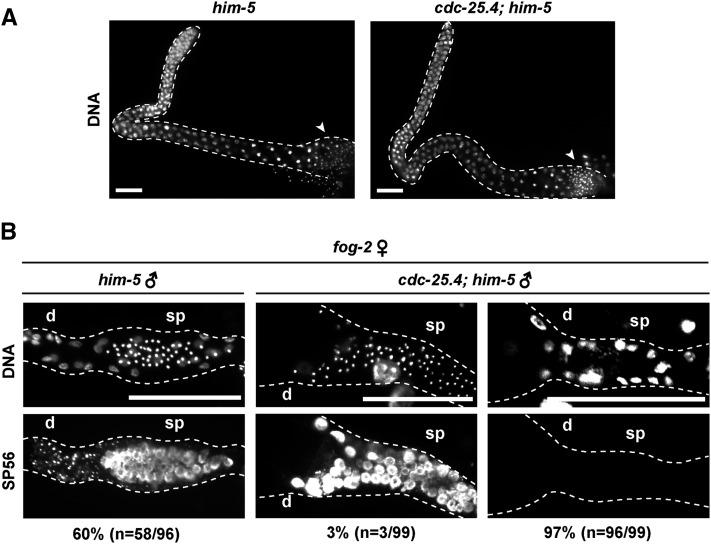
The majority of *cdc-25.4*(*tm4088*) males failed to transfer sperm to hermaphrodite uteruses. (A) DNA staining of male adult gonads. *him-5*(*e1467*) (*n* = 45) and *cdc-25.4*(*tm4088*); *him-5*(*e1467*) (*n* = 57) male adults were dissected and their gonads were stained with a DNA fluorescent dye, Hoechst 33342. Spermatids (arrowheads) were observed normally in the proximal region of *cdc-25.4*(*tm4088*); *him-5*(*e1467*) male gonads as compared with *him-5*(*e1467*) male gonads. Scale bars, 20 μm. (B) After mating with either single *him-5*(*e1467*) males or single *cdc-25.4*(*tm4088*); *him-5*(*e1467*) males, each *fog-2*(*q71*) hermaphrodite was dissected and the proximal region of the gonad was immunostained with a sperm-specific monoclonal antibody, SP56, along with Hoechst 33342 to visualize DNA. Scale bars, 50 μm. d, distal side of the gonad; sp, spermatheca.

To determine whether sperm are produced normally in *cdc-25.4* mutants, we examined male gonad arms after DNA staining. We found that the overall gonadal morphology of *cdc-25.4*(*tm4088*); *him-5*(*e1467*) males was indistinguishable from that of *him-5*(*e1467*) control males (Figure 2A), and that similar numbers of spermatids were observed in the proximal region of *cdc-25.4*; *him-5* male gonads as in *him-5* control male gonads (Figure 2A, arrowheads). Wild-type male gonad arms consist of two regions, the distal mitotic region and the proximal meiotic region. After germ cells proliferate in the distal region by mitotic division, they move to the proximal region and begin meiosis, and spermatogenesis occurs during the course of meiotic divisions I and II to produce spermatids. Finally, immotile spermatids are transformed into motile spermatozoa through a process called spermiogenesis or sperm activation (L’Hernault 2006). To determine whether germ cells in *cdc-25.4*; *him-5* male gonads undergo normal spermatogenesis, we examined meiotic division processes during spermatogenesis by immunofluorescence analysis using an anti-α-tubulin antibody, which detects microtubules, and an anti-phospho histone H3 antibody, a metaphase marker. We found that meiotic divisions I and II occurred normally during spermatogenesis in *cdc-25.4*; *him-5* male gonads as in *him-5* control male gonads (Figure S2A). In *C. elegans*, male spermatids are activated when they are transferred to hermaphrodite uteri (Ward *et al.* 1983). Spermatids recovered from male gonads can be activated *in vitro* to become motile spermatozoa by treatment with several agents, including pronase (Ward *et al.* 1983). We found that spermatids obtained from *cdc-25.4*; *him-5* male gonads were successfully activated *in vitro* after pronase treatment, as were spermatids from *him-5* control male gonads (Figure S2, B and C). These results indicate that spermatogenesis (meiotic divisions) and spermiogenesis (sperm activation) occur normally in *cdc-25.4* mutant male gonads, and thus, *cdc-25.4* mutant males can produce functional sperm.

### cdc-25.4 is required for successful male mating

*C. elegans* males deliver their sperm to hermaphrodites through a complicated male-specific mating behavior. Since sperm production in *cdc-25.4* mutant males was judged to be normal, we next asked whether the sperm of *cdc-25.4* mutant males could be successfully transferred to hermaphrodites. To answer this question, we monitored sperm transfer by immunostaining *fog-2*(*q71*) hermaphrodite gonads with a sperm-specific monoclonal SP56 antibody after *fog-2* hermaphrodites were mated with *cdc-25.4* mutant males. We found that 97% of the *fog-2* hermaphrodites failed to display sperm-specific SP56 signals after being mated with *cdc-25.4*; *him-5* males, while 60% of the *fog-2* hermaphrodites showed strong SP56 signals in their spermatheca after being mated with *him-5* control males (Figure 2B). These results indicate that *cdc-25.4* is required for successful sperm transfer, but not for sperm development or sperm activation. Therefore, when sperm were successfully transferred from a *cdc-25.4* mutant male to a hermaphrodite, which occurred at a low frequency, that hermaphrodite could produce substantial numbers of outcrossed progeny (Figure 1D).

**Figure 3:**
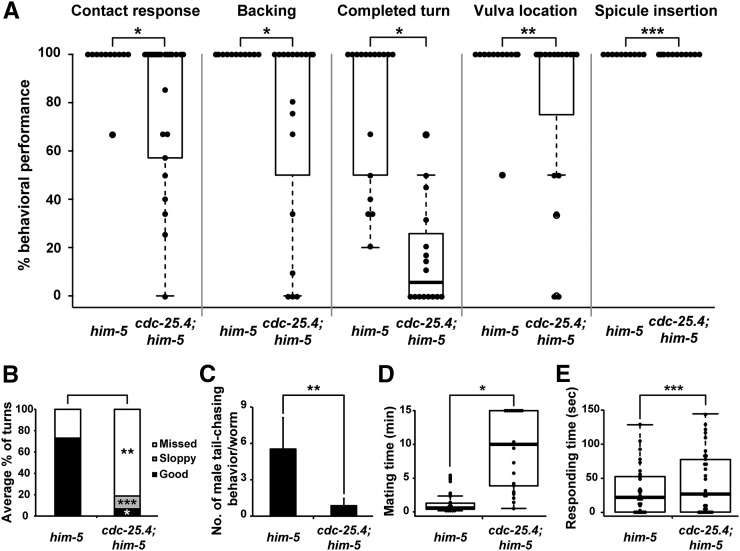
*cdc-25.4* functions in male mating behavior. (A) Percent behavioral performance in respective mating processes by *him-5*(*e1467*) males (*n* = 23) and by *cdc-25.4*(*tm4088*); *him-5*(*e1467*) males (*n* = 21). Male mating behavior was subdivided into the following processes: contact response, backing, completed turn, vulva location, and spicule insertion. Distribution of percent performance in the different processes displayed by each genotype is shown as a box plot. (B) Percent distribution of performance in turning behavior by *him-5*(*e1467*) males and by *cdc-25.4*(*tm4088*); *him-5*(*e1467*) males. Performance of turning behavior was classified into three levels: good, sloppy, and missed. (C) *cdc-25.4*(*tm4088*) mutant males showed defective tail-chasing behavior. The number of successful male tail-chasing incidents was scored in *him-5*(*e1467*) males (*n* = 17) and *cdc-25.4*(*tm4088*); *him-5*(*e1467*) males (*n* = 30) for 30 min. (D) Distribution of the time period (min) required for each mating event by *him-5*(*e1467*) males and by *cdc-25.4*(*tm4088*); *him-5*(*e1467*) males, displayed as a box plot. (E) Distribution of the time (sec) required to respond to hermaphrodites by *him-5*(*e1467*) males and by *cdc-25.4*(*tm4088*); *him-5*(*e1467*) males is displayed as a box plot. Box plots were created using *R*. Horizontal lines represent minimum, lower quartile (25%), median (50%), upper quartile (75%), and maximum, from the bottom to the top, in the box plot. Outliers represent less or more than two-thirds of the quartile. Dot indicates individual data points. Error bars indicate SD. *P* values were calculated by Student’s *t*-test against *him-5* controls. * *P* < 0.005, ** *P* < 0.05, and *** *P* > 0.05.

To further elucidate the roles of *cdc-25.4* in male mating behavior, we attempted to determine in which step of mating behavior *cdc-25.4* mutant males display a defect. Mating behavior can be subdivided into a series of processes, beginning with contact response, proceeding to backing, turning, and vulva location, then ending with spicule insertion (Loer and Kenyon 1993; Koo *et al.* 2011). We found that *cdc-25.4*; *him-5* males showed defects in most of these steps except spicule insertion, compared with control *him-5* males (Figure 3A). Among the mating processes, turning behavior was most severely affected in *cdc-25.4* mutant males (*P* < 0.005). When defects in turning behavior were further divided into several classes according to the degree of the defect, we noticed that only 4.7% of the observed *cdc-25.4*; *him-5* males showed “good” turning behavior (Figure 3B). That is, only 4.7% of tested *cdc-25.4* mutant males could successfully turn without losing contact between their tails and the bodies of the hermaphrodites (movie File S1 and movie File S2). It was reported that appropriate tail curling is necessary for good male turning (Loer and Kenyon 1993). Tail curling is also required when a male makes a circular backward movement, which occurs when he chases his own head with his tail. This behavior is called “male tail-chasing behavior” (Wang *et al.* 2014). To determine whether *cdc-25.4* mutant males show a defect in tail-chasing behavior, we scored how many times a *cdc-25.4* mutant male successfully performed the tail-chasing behavior in a 30 min time interval (see *Materials and Methods*). We found that *cdc-25.4*; *him-5* males performed significantly lower numbers of tail-chasing behavior compared to *him-5* control males (*P* < 0.05) (Figure 3C). This result suggests that *cdc-25.4* mutant males show a significant defect in turning behavior because their tails cannot curl well. We next measured the time required for mating. We defined this as a time interval required since each male was placed on a mating plate until he finished ejaculation. When mating did not occur or did not complete, we stopped the observation after 15 min elapsed, and considered the elapsed time of the trial to be 15 min. By this assay, we found that the time required for mating was significantly longer for *cdc-25.4*; *him-5* males than for *him-5* control males (*P* < 0.001) (Figure 3D). We also found that only when *cdc-25.4*; *him-5* males contacted the ventral side of hermaphrodites by chance, could they locate the vulva and finish mating in a short period of time, possibly because they did not have to turn. By contrast, we found that responding time, which is the time interval since a male was placed on a mating plate until the male made his first successful contact with a hermaphrodite, was not significantly different between *cdc-25.4*; *him-5* males and *him-5* control males (*P* > 0.05) (Figure 3E). This result suggests that the longer mating time observed for *cdc-25.4* mutant males was due to a defect that occurred after contacting hermaphrodites, most likely a defect in turning behavior. Taken together, these results indicate that the low fertility of *cdc-25.4* mutant males was due to behavioral defects in several steps of the male mating processes.

### cdc-25.4 is expressed in many neuronal cells and germ cells

**Figure 4:**
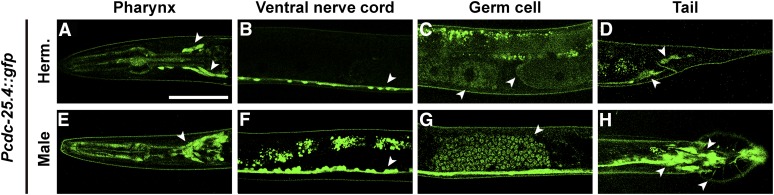
*Pcdc-25.4*::*gfp* is expressed in many neurons and germ cells. The *Pcdc-25.4*::*gfp* transgene was expressed in the pharynx (A, E), ventral nerve cord (B, F), oocytes (C), sperm (C, G), and tail neurons (D, H), in the transgenic hermaphrodites (Herm.) and males. Arrowheads indicate the loci where *Pcdc-25.4*::*gfp* was detected. Scale bar, 50 μm.

To determine the expression pattern of *cdc-25.4*, we generated several lines of transgenic animals carrying a *Pcdc-25.4*::*gfp* reporter construct. Surprisingly, we found that *Pcdc-25.4*::*gfp* was expressed in many neurons, including pharyngeal neurons (Figure 4, A and E), the ventral nerve cord (Figure 4, B and F), and tail neurons (Figure 4, D and H) in both hermaphrodites and males. However, the signal was not detected in the dorsal nerve cord. *Pcdc-25.4*::*gfp* expression was also observed in pharyngeal muscles, the head ganglion, preanal ganglion, and lumbar ganglion. In particular, *Pcdc-25.4*::*gfp* was expressed in ray cell bodies, spicule neurons, hook neurons, and postcloacal sensilla neurons in male tails (Figure S3). *cdc-25.4* was also expressed in germ cells, including oocytes and sperm (Figure 4, C and G). We also found that *Pcdc-25.4*::*gfp* was expressed in many neurons throughout larval development (Figure S4). Neuronal expression of *cdc-25.4* was confirmed by colocalization of a pan-neuronal marker, *Prab-3*::*mCherry* (Figure S4).

### Turning defect of cdc-25.4 mutant males was rescued by pan-neuronal expression of a cdc-25.4 transgene

**Figure 5:**
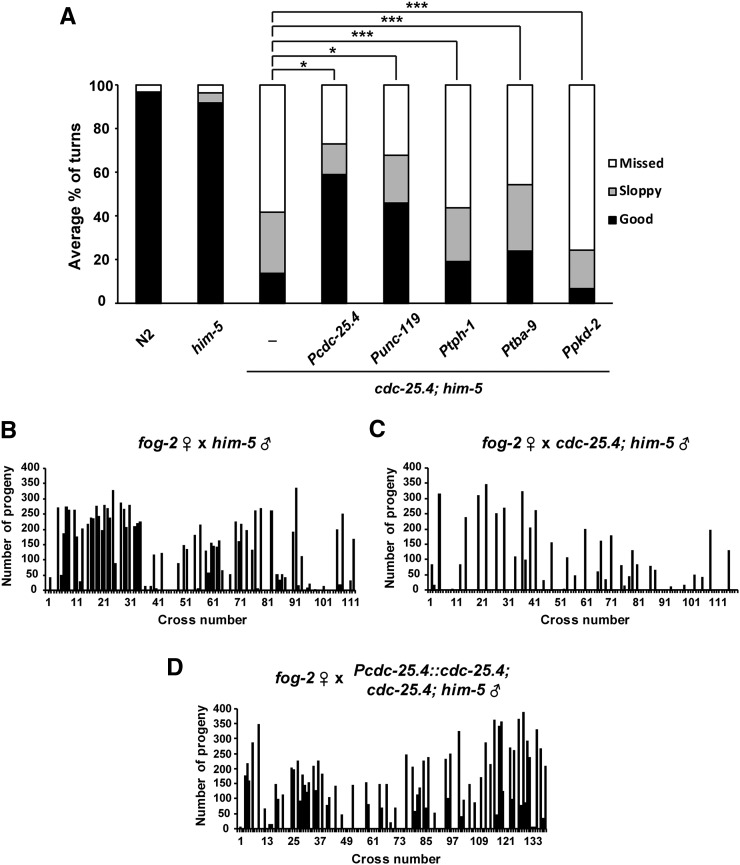
The rescue of defective phenotypes in *cdc-25.4*(*tm4088*) males. (A) The turning behavior in *cdc-25.4*(*tm4088*) males was rescued by a pan-neuronal promoter-driven *cdc-25.4* transgene. Possible rescue of defective turning behavior in *cdc-25.4*(*tm4088*) males by several neuron-specific promoter-driven *cdc-25.4* transgenes was tested. Levels of turning behavior were classified into good, sloppy, and missed. Percent distribution of the respective turning behavior levels were measured in N2 (*n* = 21), *him-5*(*e1467*) (*n* = 59), and *cdc-25.4*(*tm4088*); *him-5*(*e1467*) (*n* = 61) males, as well as in *cdc-25.4*(*tm4088*); *him-5*(*e1467*) males harboring a *gfp*::*cdc-25.4* transgene driven by either the *cdc-25.4* (positive control, *n* = 21), *unc-119* (expressed pan-neuronally, *n* = 14), *tph-1* (expressed in serotonergic neurons, *n* = 21), *tba-9* (expressed in ray A neurons, *n* = 20), or *pkd-2* (expressed in ray B neurons, *n* = 8) promoters. (B) The number of progeny produced by *fog-2*(*q71*) single hermaphrodites after mating with single *him-5*(*e1467*) males (*n* = 113). 64.6% of the *fog-2*(*q71*) hermaphrodite parents produced outcrossed progeny. (C) The number of progeny produced by *fog-2*(*q71*) single hermaphrodites after mating with single *cdc-25.4*(*tm4088*); *him-5*(*e1467*) males (*n* = 119). 68.1% of the *fog-2*(*q71*) hermaphrodite parents failed to produce outcrossed progeny. (D) The number of progeny produced by *fog-2*(*q71*) single hermaphrodites after mating with single *Pcdc-25.4*::*cdc-25.4*; *cdc-25.4*(*tm4088*); *him-5*(*e1467*) males (*n* = 140). 54.3% of the *fog-2*(*q71*) hermaphrodite parents produced outcrossed progeny. *P* values were calculated by Student’s *t*-test. * *P* < 0.001 and *** *P* > 0.05.

To test whether neuronal expression of a *cdc-25.4* transgene can rescue the defect in turning behavior seen in *cdc-25.4* mutant males, we generated several *cdc-25.4* transgene constructs in which different promoters controlled the expression of *cdc-25.4*. These *cdc-25.4* transgenes were examined to see which promoter-driven transgene could successfully rescue the turning defect of *cdc-25.4* mutant males. First, we confirmed that *cdc-25.4*’*s* own promoter-driven *cdc-25.4* transgene significantly rescued the turning defect of *cdc-25.4* mutant males (*P* < 0.001) (Figure 5A). The fertility defect of *cdc-25.4* mutant males was also rescued by *cdc-25.4* promoter-driven *cdc-25.4* transgene expression (Figure 5, B–D). Furthermore, we found that a *cdc-25.4* transgene driven by the *unc-119* promoter, which is expressed pan-neuronally, could significantly rescue the turning defect of *cdc-25.4*; *him-5* males (*P* < 0.001) (Figure 5A), indicating that *cdc-25.4* is required in neurons for successful male turning.

It is known that male turning behavior, the most prominent defective phenotype in *cdc-25.4* mutant males, is mediated by 5-HT and DA (Loer and Kenyon 1993; Liu and Sternberg 1995). Exogenous 5-HT treatment led to an increase in male tail curling behavior (Loer and Kenyon 1993). In addition, ablation of serotonergic CP motor neurons, which are located in the male ventral nerve cord, and 5-HT-deficient mutant males exhibited reduced turning behavior (Loer and Kenyon 1993). In addition, the R5A, R7A, and R9A neurons, which release DA, are required for tail ventral turning, and ablation of these neurons in males results in sloppy turning behavior (Sulston *et al.* 1975; Sulston and Horvitz 1977; Liu and Sternberg 1995). Based on these previous studies, we questioned whether *cdc-25.4* is involved in either synthesis or secretion of these neurotransmitters. To test this possibility, *cdc-25.4* mutant males were exogenously treated with neurotransmitters. However, we found that exogenous DA failed to rescue the turning defect of *cdc-25.4*; *him-5* males (Figure S5A). Furthermore, 5-HT treatment also failed to rescue the turning defect of *cdc-25.4*; *him-5* males (Figure S5B). These results suggest either that *cdc-25.4* does not have an essential function in serotonergic or dopaminergic neurons or that *cdc-25.4* is not required for the synthesis or secretion of these neurotransmitters.

Although exogenous treatment of 5-HT and DA failed to rescue the turning defect of *cdc-25.4* mutant males, to confirm that *cdc-25.4* does not have an essential function in serotonergic or dopaminergic neurons, we tested whether a *cdc-25.4* transgene driven by the *tph-1* promoter or the *dat-1* promoter could rescue the turning defect of *cdc-25.4* mutant males. *tph-1* and *dat-1* are specifically expressed in serotonergic and dopaminergic neurons, respectively. *tph-1* encodes a tryptophan hydroxylase that is required for 5-HT biosynthesis (Sze *et al.* 2000). *dat-1* encodes a DA transporter and controls dopaminergic neurotransmission (Jayanthi *et al.* 1998; Nass *et al.* 2002). We found that expression of a *cdc-25.4* transgene under the control of the *tph-1* promoter could not rescue the turning defect of *cdc-25.4* mutant males to a significant degree (*P* > 0.05). This result suggests that expression of *cdc-25.4* in serotonergic neurons alone is not sufficient to rescue the *cdc-25.4* male mutant phenotype (Figure 5A). Because we failed to obtain transgenic animals in which a *cdc-25.4* transgene is expressed under the control of the *dat-1* promoter, we could not determine whether expression of *cdc-25.4* in dopaminergic neurons alone is sufficient to rescue the *cdc-25.4* male mutant phenotype. We suspect that *cdc-25.4* expression under the control of the *dat-1* promoter may induce some toxic effects. In place of the *dat-1* promoter, we tried to express *cdc-25.4* transgenes under the control of the *tba-9* promoter and the *pkd-2* promoter to express *cdc-25.4* in ray A-type and B-type neurons, respectively. *tba-9* is one of the nine α tubulins and is expressed in various ciliated sensory neurons, including several male-specific neurons (Gogonea *et al.* 1999; Hurd *et al.* 2010). *pkd-2* is expressed in male-specific sensory neurons and required for male mating behavior (Barr and Sternberg 1999; Barr *et al.* 2001). We found that *cdc-25.4* transgene expression in ray A-type neurons under the control of the *tba-9* promoter rescued the turning defect of *cdc-25.4* mutant males slightly, but not significantly (*P* > 0.05) (Figure 5A). It must be noted that *tba-9* expression is not limited to ray A-type neurons. It is also expressed in other ciliated neurons that are located in the head and ventral nerve cord (Hurd *et al.* 2010). The *tba-9* promoter-driven *cdc-25.4* expression in multiple ciliated neurons might have contributed to the slight rescue of the defective turning behavior. By contrast, *cdc-25.4* transgene expression under the control of the *pkd-2* promoter completely failed to rescue the turning defect of *cdc-25.4* mutant males. These results suggest that *cdc-25.4* would rather function in multiple neurons than a subset of serotonergic, dopaminergic, or ray neurons.

### The neuronal morphology of cdc-25.4 mutant males is generally normal

Since *cdc-25.4* is expressed in many neurons and considered to be an evolutionarily conserved cell division cycle regulator, we questioned whether *cdc-25.4* affects neuronal cell divisions in males. If neuronal cell divisions in *cdc-25.4* mutant males are defective, this defect most likely results in abnormal numbers of neurons, and thus, abnormal neuronal morphology. To test this possibility, we compared gross neuronal morphology between *him-5* control adult males and *cdc-25.4*; *him-5* adult males under fluorescence microscopy after introducing a pan-neuronally expressing *Punc-119*::*gfp* transgene to these males (Figure S6). Despite careful examination, we could not detect any obvious difference in the neuronal expression pattern of the *Punc-119*::*gfp* transgene between *him-5* control males and *cdc-25.4*; *him-5* males.

**Figure 6:**
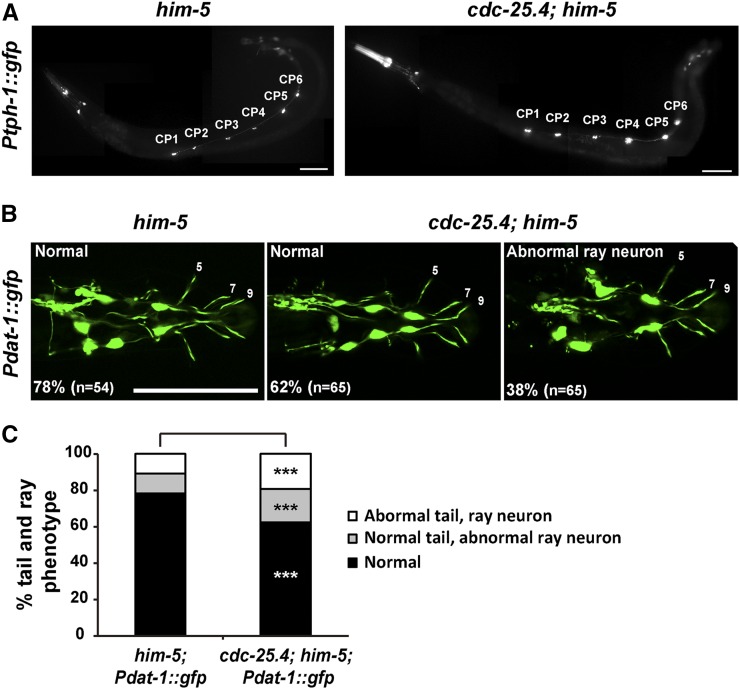
The number and morphology of serotonergic CP neurons and dopaminergic ray neurons appear normal in *cdc-25.4* mutant males. (A) Male-specific CP1–6 neurons, which were labeled with the *Ptph-1*::*gfp* transgene, were detected normally on the ventral nerve cord in both *him-5*(*e1467*) and *cdc-25.4*(*tm4088*); *him-5*(*e1467*) males. (B) The number and morphology of dopaminergic ray neurons (R5A, R7A, and R9A), which were labeled with the *Pdat-1*::*gfp* transgene, were not significantly different between *him-5*(*e1467*) and *cdc-25.4*(*tm4088*); *him-5*(*e1467*) males. (C) Percent distribution of male tail phenotypes in *him-5*(*e1467*); *Pdat-1*::*gfp* and *cdc-25.4*(*tm4088*); *him-5*(*e1467*); *Pdat-1*::*gfp* transgenic males. Male tail phenotypes were classified into three classes: normal tail morphology with normal DA ray neurons, normal tail morphology with abnormal DA ray neurons, and abnormal tail morphology with abnormal DA ray neurons. Scale bars, 50 μm. *P* values were calculated by Student’s *t*-test against *him-5*; *Pdat-1*::*gfp* controls. *** *P* > 0.05. DA, dopamine.

Although *cdc-25.4* transgene expression in serotonergic and dopaminergic neurons under the control of the *tph-1* promoter and the *dat-1* promoter, respectively, could not rescue the turning defect of *cdc-25.4* mutant males, to determine whether *cdc-25.4* mutant males have any morphological defects in these neurons, we examined the morphology of serotonergic CP motor neurons and dopaminergic R5A, R7A, and R9A neurons after introducing *Ptph-1*::*gfp* and *Pdat-1*::*gfp* transgenes into *cdc-25.4* mutant males. We found that CP 1-6 motor neurons were morphologically normal in *cdc-25.4*; *him-5* males as compared to *him-5* control males (Figure 6A). On the other hand, we found that *cdc-25.4*; *him-5* males showed slight abnormalities in ray neuronal morphology (38%, *n* = 65) as compared to *him-5* control males (22%, *n* = 54), although the difference was not significant (*P* > 0.05) (Figure 6, B and C). These results suggest that the defect in turning behavior in *cdc-25.4* mutant males is most likely not caused by a loss of neurons or abnormal neuronal morphology.

### cdc-25.4 may function in a noncanonical manner in male neurons to regulate male mating

**Figure 7:**
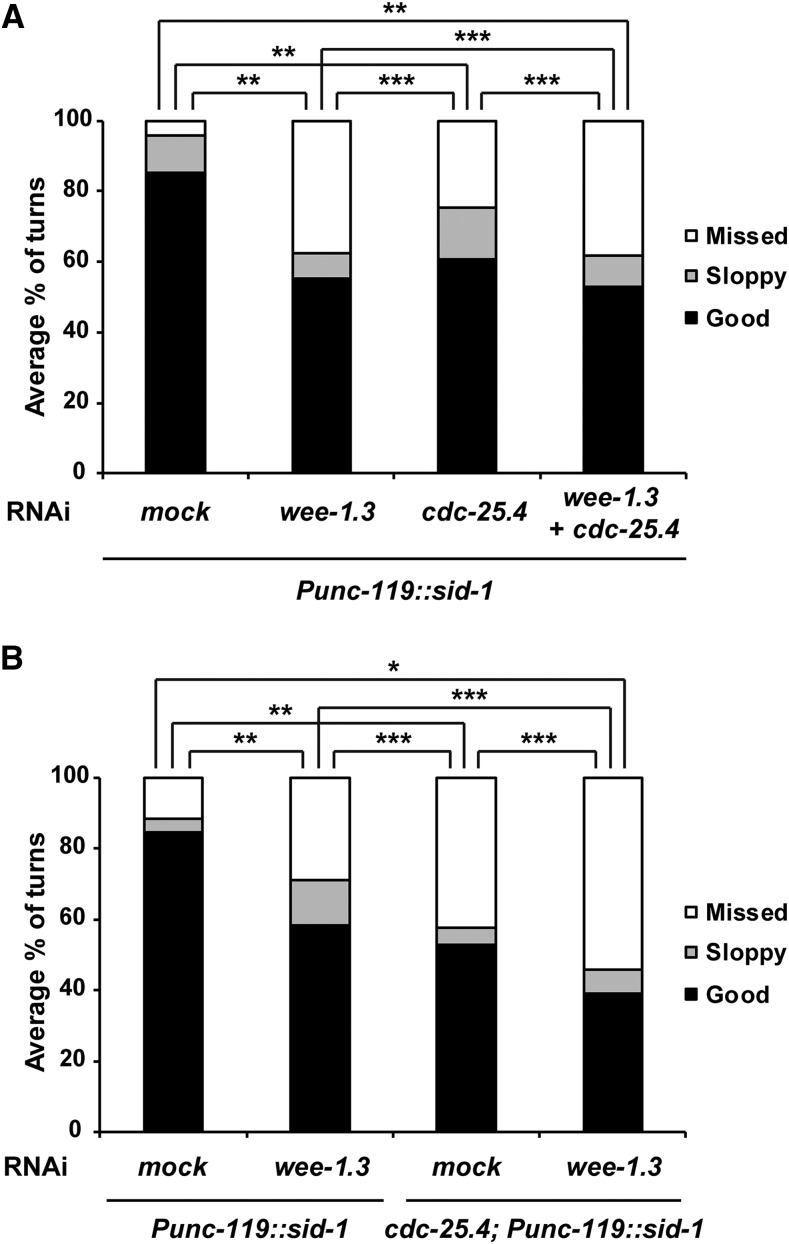
RNAi depletion of *wee-1.3* did not suppress defective turning behavior of *cdc-25.4*(*tm4088*) males. (A) Levels of turning behavior in *Punc-119*::*sid-1* neuron-specific RNAi males, which were classified into good, sloppy, and missed, were measured after either mock (*n* = 16), *wee-1.3* (*n* = 18), *cdc-25.4* (*n* = 22), or *wee-1.3* plus *cdc-25.4* double (*n* = 13) RNAi depletion at the L1 larval stage. (B) Levels of turning behavior in *Punc-119*::*sid-1* and *cdc-25.4*(*tm4088*); *Punc-119*::*sid-1* males were measured after either mock (*n* = 18) or *wee-1.3* (*n* = 19) RNAi depletion at the L1 larval stage. *P* values were calculated by Student’s *t*-test against mock RNAi. * *P* < 0.001, ** *P* < 0.05, and *** *P* > 0.05. RNAi, RNA interference.

In canonical cell division cycle regulation, Wee1 kinase and Cdc25 phosphatase compete against each other to regulate Cdk1 activity (Fantes 1979). That is, Wee1 kinase inactivates Cdk1 by phosphorylating specific amino acid residues on Cdk1, and Cdc25 phosphatase activates Cdk1 by removing inhibitory phosphates from these residues (Hagan and Grallert 2013). We previously reported that *cdc-25.1* and *cdc-25.2*, members of the *C. elegans cdc-25* family, counteract *wee-1.3* in germ cell proliferation, oocyte maturation, and intestinal division (Kim *et al.* 2010; Yoon *et al.* 2012; Lee *et al.* 2016). Three *wee-1* genes have been identified in *C. elegans*; *wee-1.1*, *wee-1.2*, and *wee-1.3*. *wee-1.1* is only expressed in the E cell of the 12-cell-stage embryo, which develops into the intestine. *wee-1.2* is a pseudogene that is not transcribed (Wilson *et al.* 1999; Robertson *et al.* 2014). *wee-1.3* is expressed in the embryos, adult soma, and germline (Lamitina and L’Hernault 2002; Allen *et al.* 2014). To determine whether *wee-1.3* also counteracts *cdc-25.4* in neurons during male mating, we examined the male turning behavior of *cdc-25.4* mutants with or without RNAi depletion of *wee-1.3*. We found that *wee-1.3* RNAi did not suppress the turning defect of *cdc-25.4* mutant males (Figure S7A). Instead, we found that *wee-1.3* RNAi single-handedly caused a severe turning defect in *him-5* control males. Furthermore, mating time was significantly extended after *wee-1.3* RNAi treatment in *him-5* control males (Figure S7B). We also observed that rays were abolished in male tails when *wee-1.3* alone was depleted by RNAi (Figure S7C). Depletion of *wee-1.3* caused severe defects not only in neuronal cells, but also in other types of cells. These cellular defects might be the cause of defective male mating in *wee-1.3* RNAi-treated animals. To determine the function of *wee-1.3* in neurons, we performed neuron-specific RNAi depletion of *wee-1.3* using a strain, TU3311: *Punc-119*::*sid-1*, in which neuronal RNAi is specifically enhanced. This strain expresses the *sid-1*(*+*) transgene in all the neuronal cells by the *unc-119* promoter (Calixto *et al.* 2010), which results in enhancement of neuronal RNAi. In addition, pan-neuronal expression of *sid-1* reduces RNAi efficiency in nonneuronal tissues. We found that RNAi depletion of *wee-1.3* in *Punc-119*::*sid-1* worms caused significant defects in male turning behavior (Figure 7A). On the other hand, this neuron-specific *wee-1.3* RNAi did not cause any significant defects in male tail development (Figure S7C), suggesting that *wee-1.3* specifically functions in neuronal cells to control male mating behavior. We also confirmed that neuron-specific *cdc-25.4* RNAi in *Punc-119*::*sid-1* worms caused significant defects only in male turning behavior (Figure 7A). However, double RNAi depletion of *wee-1.3* and *cdc-25.4* in *Punc-119*::*sid-1* worms did not suppress the turning defects observed in the *cdc-25.4* single RNAi-treated *Punc-119*::*sid-1* worms (Figure 7A). To confirm the relationship between *cdc-25.4* and *wee-1.3* in mating behavior, we introduced *cdc-25.4* deletion mutation into the *Punc-119*::*sid-1* worms, and tested the turning behavior after *wee-1.3* RNAi treatment. We found that neuron-specific *wee-1.3* RNAi did not suppress the turning defects of *cdc-25.4* mutant males (Figure 7B), suggesting that *wee-1.3* and *cdc-25.4* are not competing against each other for the control of male turning behavior. Therefore, *cdc-25.4* and *wee-1.3* likely function independently for the control of male mating. These results also imply that, unlike other *cdc-25* family members, *cdc-25.4* may play a noncanonical role in neurons, which is distinct from canonical cell cycle regulation.

## Discussion

In this study, we found that *cdc-25.4* is required for male fertility through controlling the mating behavior. It appears that *cdc-25.4* does not function only in a single step during the mating processes, because *cdc-25.4* mutant males showed behavioral defects in various processes, including contact response, backing, turning, and vulva location, but not in spicule insertion (Figure 3A). In addition, *cdc-25.4* is expressed in many neuronal cells, like the pan-neuronal gene *rab-3* (Figure S4). Transgenic expression of *cdc-25.4*, driven either by its own promoter or by the pan-neuronal *unc-119* promoter, rescued the *cdc-25.4* mutant males’ mating ability. However, *cdc-25.4* transgenes under the control of promoters that are expressed in specific types of neurons failed to rescue the *cdc-25.4* mutant male phenotypes (Figure 5A). These results suggest that *cdc-25.4* has multiple functions in neurons and that it is required pan-neuronally for male fertility. It took much longer time periods for the mutant males to locate vulva and ejaculate sperm, because *cdc-25.4* mutant males are defective in contact response, backing, and turning, which are necessary for finding vulva and ejaculating sperm (Figure 3, A–D). However, response time to hermaphrodites was not delayed in *cdc-25.4* mutant males, suggesting that they are not defective in searching for hermaphrodites (Figure 3E).

Although the *cdc-25.4* mRNA expression level was high in the L4 larval stage in hermaphrodites, when spermatogenesis occurs, and *Pcdc-25.4*::*gfp* was expressed in sperm, neither spermatogenesis nor spermiogenesis were impaired in both *cdc-25.4* mutant males and hermaphrodites (Figure S2). Unexpectedly, we did not observe any obvious defects in hermaphrodite or male germlines, suggesting that the other members of the *cdc-25* gene family compensate for the function of *cdc-25.4* in the germline, unlike in neuronal cells. All four members of the *C. elegans cdc-25* gene family, *cdc-25.1* (Ashcroft *et al.* 1999; Ashcroft and Golden 2002; Kim *et al.* 2009; Yoon *et al.* 2012), *cdc-25.2* (Kim *et al.* 2010), *cdc-25.3* (unpublished data), and *cdc-25.4* (Figure 1A), are highly expressed in the germline. Therefore, they are expected to have functions in the germline. However, unlike other family members, we did not observe any obvious germline defects in *cdc-25.4* mutants. Considering the expression patterns of the *cdc-25* gene family, possible germline function of *cdc-25.4* can be redundant with that of *cdc-25.1* in the male germline (Ashcroft *et al.* 1999), and with that of *cdc-25.1*, *cdc-25.2*, and *cdc-25.3* in the hermaphrodite germline. This possibility remains to be elucidated. While the expression level of *cdc-25.4*, which was quantified by qRT-PCR, suggested that *cdc-25.4* is abundantly expressed in the germline (Figure 1A), the *cdc-25.4* transgene driven by its own promoter was mainly expressed in neuronal cells including the male-specific neurons. It is known that male-specific neurons are formed during the L3 and L4 larval stages by growing axons and dendrites (Sulston *et al.* 1980). The fact that the highest expression of *cdc-25.4* was observed at the L4 larval stage supports the idea that activity of *cdc-25.4* is required for male neuronal cell functions.

The roles of *C. elegans cdc-25* family genes in cell cycle progression in the germline (Ashcroft *et al.* 1999; Ashcroft and Golden 2002; Kim *et al.* 2009, 2010; Yoon *et al.* 2012), and in the intestine (Hebeisen and Roy 2008; Segref *et al.* 2010; Lee *et al.* 2016) have been previously reported. However, in this study, we revealed a noncanonical function of *cdc-25.4* that is uncoupled from cell cycle regulation, based on the following three observations. (1) neuron-specific depletion of *wee-1.3*, a *C. elegans* ortholog of Wee1/Myt1 kinase, in *cdc-25.4* mutant males did not suppress the defective phenotypes of *cdc-25.4* mutant males. WEE-1.3 inactivates cyclin-dependent kinases by adding inhibitory phosphates, which are removed by CDC-25 phosphatase to promote the cell cycle (Fantes 1979; Russell and Nurse 1987). Thus, *wee-1.3* normally counteracts *cdc-25* in canonical cell cycle regulation. (2) The expression patterns of the *Punc-119*::*gfp* transgene in neuronal cells were indistinguishable between *cdc-25.4* mutants and the wild type. (3) *cdc-25.4* was expressed in already differentiated postmitotic neurons. These observations suggest that *cdc-25.4* is required for neuronal function rather than neuronal development. Noncanonical functions of some cell cycle regulators have been previously reported. CDK7, CDK8, and CDK9 regulate transcription in humans (Malumbres 2014). Cell cycle-independent roles of CDK5 have been identified in a mammalian nervous system (Su and Tsai 2011). Interestingly, *C. elegans* CDK-5 is also required for neuronal functions (Ou *et al.* 2010; Park *et al.* 2011; Goodwin *et al.* 2012; Monteiro *et al.* 2012). Further, *C. elegans* CDK-8 regulates axon navigation decisions in the nervous system (Steimel *et al.* 2013). These studies support the idea that functions of cell cycle regulators are not limited to the cell cycle regulation. We should rather consider that they have the potential to execute noncanonical functions.

The most prominent behavioral defect observed in *cdc-25.4* mutant males was the turning defect. Previous studies indicate that the male-specific CP ventral cord motor neurons (Loer and Kenyon 1993) and the three posterior-most rays, rays 7–9, are involved in male turning behavior (Liu and Sternberg 1995). Ablation of CP and ray 7–9 neurons caused defects in turning behavior (Loer and Kenyon 1993; Liu and Sternberg 1995). The CP neurons control tail moving by forming synapses with the male-specific diagonal muscles (White 1988). 5-HT and DA neurotransmitters were previously reported to be involved in male turning behavior (Loer and Kenyon 1993; Liu and Sternberg 1995). However, we found that the defective turning behavior in *cdc-25.4* mutant males was not caused by a failure in the synthesis or secretion of these neurotransmitters, because *cdc-25.4* mutant phenotypes were not recovered when we treated the worms with 5-HT or DA (Figure S5). Furthermore, CP neurons and R5A, R7A, and R9A neurons visualized by *Ptph-1*::*gfp* and *Pdat-1*::*gfp* expression were morphologically normal in *cdc-25.4* mutant males. This observation suggests that *cdc-25.4* is not involved in the formation of these neurons (Figure 6). In summary, we identified a novel noncanonical function of CDC-25.4 phosphatase in neuronal cells that is required for successful male mating behavior.

## Supplementary Material

Supplemental Material

## References

[bib1] Allen A. K. Nesmith J. E. Golden A., 2014 An RNAi-based suppressor screen identifies interactors of the Myt1 ortholog of *Caenorhabditis elegans*. G3 (Bethesda) 4: 2329–2343. 25298536 10.1534/g3.114.013649 PMC4267929

[bib2] Ashcroft N. Golden A., 2002 CDC-25.1 regulates germline proliferation in *Caenorhabditis elegans*. Genesis 33: 1–7. 12001064 10.1002/gene.10083

[bib3] Ashcroft N. R. Kosinski M. E. Wickramasinghe D. Donovan P. J. Golden A., 1998 The four *cdc25* genes from the nematode *Caenorhabditis elegans*. Gene 214: 59–66. 9651482 10.1016/s0378-1119(98)00228-5

[bib4] Ashcroft N. R. Srayko M. Kosinski M. E. Mains P. E. Golden A., 1999 RNA-mediated interference of a *cdc25* homolog in *Caenorhabditis elegans* results in defects in the embryonic cortical membrane, meiosis, and mitosis. Dev. Biol. 206: 15–32. 9918692 10.1006/dbio.1998.9135

[bib5] Austin J. Kimble J., 1987 *glp-1* is required in the germ line for regulation of the decision between mitosis and meiosis in *C. elegans*. Cell 51: 589–599. 3677168 10.1016/0092-8674(87)90128-0

[bib6] Barr, M. M., and L. R. Garcia, 2006 Male mating behavior (June 19, 2006). *WormBook*, ed. The *C. elegans* Research Community WormBook, 10.1895/wormbook.1.78.1, http://www.wormbook.org. 10.1895/wormbook.1.78.1 PMC4780960 18050467

[bib7] Barr M. M. Sternberg P. W., 1999 A polycystic kidney-disease gene homologue required for male mating behaviour in *C. elegans*. Nature 401: 386–389. 10517638 10.1038/43913

[bib8] Barr M. M. DeModena J. Braun D. Nguyen C. Q. Hall D. H., 2001 The *Caenorhabditis elegans* autosomal dominant polycystic kidney disease gene homologs *lov-1* and *pkd-2* act in the same pathway. Curr. Biol. 11: 1341–1346. 11553327 10.1016/s0960-9822(01)00423-7

[bib9] Barton M. K. Schedl T. B. Kimble J., 1987 Gain-of-function mutations of *fem-3*, a sex-determination gene in *Caenorhabditis elegans*. Genetics 115: 107–119. 3557107 10.1093/genetics/115.1.107 PMC1203045

[bib10] Brenner S., 1974 The genetics of *Caenorhabditis elegans*. Genetics 77: 71–94. 4366476 10.1093/genetics/77.1.71 PMC1213120

[bib11] Calixto A. Chelur D. Topalidou I. Chen X. Chalfie M., 2010 Enhanced neuronal RNAi in *C. elegans* using SID-1. Nat. Methods 7: 554–559. 20512143 10.1038/nmeth.1463 PMC2894993

[bib12] Chen S. Bohrer L. R. Rai A. N. Pan Y. Gan L., 2010 Cyclin-dependent kinases regulate epigenetic gene silencing through phosphorylation of EZH2. Nat. Cell Biol. 12: 1108–1114. 20935635 10.1038/ncb2116 PMC3292434

[bib13] Donzelli M. Draetta G. F., 2003 Regulating mammalian checkpoints through Cdc25 inactivation. EMBO Rep. 4: 671–677. 12835754 10.1038/sj.embor.embor887 PMC1326326

[bib14] Edgar B. A. O’Farrell P. H., 1989 Genetic control of cell division patterns in the *Drosophila* embryo. Cell 57: 177–187. 2702688 10.1016/0092-8674(89)90183-9 PMC2755076

[bib15] Fantes P., 1979 Epistatic gene interactions in the control of division in fission yeast. Nature 279: 428–430. 16068179 10.1038/279428a0

[bib16] Forsburg S. L. Nurse P., 1991 Cell cycle regulation in the yeasts *Saccharomyces cerevisiae* and *Schizosaccharomyces pombe*. Annu. Rev. Cell Biol. 7: 227–256. 1809348 10.1146/annurev.cb.07.110191.001303

[bib17] Frøkjær-Jensen C. Davis M. W. Hopkins C. E. Newman B. J. Thummel J. M., 2008 Single-copy insertion of transgenes in *Caenorhabditis elegans*. Nat. Genet. 40: 1375–1383. 18953339 10.1038/ng.248 PMC2749959

[bib18] Gogonea C. B. Gogonea V. Ali Y. M. Jr. Merz K. M. Siddigui S. S., 1999 Computational prediction of the three-dimensional structures for the *Caenorhabditis elegans* tubulin family. J. Mol. Graph. Model. 17: 90–100, 126–130. 10680114 10.1016/s1093-3263(99)00025-x

[bib19] Goodwin P. R. Sasaki J. M. Juo P., 2012 Cyclin-dependent kinase 5 regulates the polarized trafficking of neuropeptide-containing dense-core vesicles in *Caenorhabditis elegans* motor neurons. J. Neurosci. 32: 8158–8172. 22699897 10.1523/JNEUROSCI.0251-12.2012 PMC3392131

[bib20] Grants J. M. Ying L. T. Yoda A. You C. C. Okano H., 2016 The mediator kinase module restrains epidermal growth factor receptor signaling and represses vulval cell fate specification in *Caenorhabditis elegans*. Genetics 202: 583–599. 26715664 10.1534/genetics.115.180265 PMC4788237

[bib21] Hagan I. M. Grallert A., 2013 Spatial control of mitotic commitment in fission yeast. Biochem. Soc. Trans. 41: 1766–1771. 24256289 10.1042/BST20130190

[bib22] Hebeisen M. Roy R., 2008 CDC-25.1 stability is regulated by distinct domains to restrict cell division during embryogenesis in *C. elegans*. Development 135: 1259–1269. 18287204 10.1242/dev.014969

[bib23] Hodgkin J., 1983 Male phenotypes and mating efficiency in *Caenorhabditis elegans*. Genetics 103: 43–64. 17246100 10.1093/genetics/103.1.43 PMC1202023

[bib24] Hurd D. D. Miller R. M. Núñez L. Portman D. S., 2010 Specific alpha- and beta-tubulin isotypes optimize the functions of sensory cilia in *Caenorhabditis elegans*. Genetics 185: 883–896. 20421600 10.1534/genetics.110.116996 PMC2907207

[bib25] Jarrell T. A. Wang Y. Bloniarz A. E. Brittin C. A. Xu M., 2012 The connectome of a decision-making neural network. Science 337: 437–444. 22837521 10.1126/science.1221762

[bib26] Jayanthi L. D. Apparsundaram S. Malone M. D. Ward E. Miller D. M., 1998 The *Caenorhabditis elegans* gene *T23G5.5* encodes an antidepressant- and cocaine-sensitive dopamine transporter. Mol. Pharmacol. 54: 601–609. 9765501

[bib27] Jimenez J. Alphey L. Nurse P. Glover D. M., 1990 Complementation of fission yeast cdc2ts and cdc25ts mutants identifies two cell cycle genes from *Drosophila*: a cdc2 homologue and string. EMBO J. 9: 3565–3571. 2120044 10.1002/j.1460-2075.1990.tb07567.x PMC552107

[bib28] Kasten M. Giordano A., 2001 Cdk10, a Cdc2-related kinase, associates with the Ets2 transcription factor and modulates its transactivation activity. Oncogene 20: 1832–1838. 11313931 10.1038/sj.onc.1204295

[bib29] Kim J. Lee A. R. Kawasaki I. Strome S. Shim Y. H., 2009 A mutation of *cdc-25.1* causes defects in germ cells but not in somatic tissues in *C. elegans*. Mol. Cells 28: 43–48. 19533027 10.1007/s10059-009-0098-8 PMC2908335

[bib30] Kim J. Kawasaki I. Shim Y. H., 2010 *cdc-25.2*, a *C. elegans* ortholog of *cdc25*, is required to promote oocyte maturation. J. Cell Sci. 123: 993–1000. 20200231 10.1242/jcs.060442

[bib31] Koo P. K. Bian X. Sherlekar A. L. Bunkers M. R. Lints R., 2011 The robustness of *Caenorhabditis elegans* male mating behavior depends on the distributed properties of ray sensory neurons and their output through core and male-specific targets. J. Neurosci. 31: 7497–7510. 21593334 10.1523/JNEUROSCI.6153-10.2011 PMC6622613

[bib32] Lamitina S. T. L’Hernault S. W., 2002 Dominant mutations in the *Caenorhabditis elegans Myt1* ortholog *wee-1.3* reveal a novel domain that controls M-phase entry during spermatogenesis. Development 129: 5009–5018. 12397109 10.1242/dev.129.21.5009

[bib33] LeBoeuf B. Correa P. Jee C. García L. R., 2014 *Caenorhabditis elegans* male sensory-motor neurons and dopaminergic support cells couple ejaculation and post-ejaculatory behaviors. eLife 3: e02938. 10.7554/eLife.02938 PMC4103683 24915976

[bib34] Lee Y. U. Son M. Kim J. Shim Y. H. Kawasaki I., 2016 CDC-25.2, a *C. elegans* ortholog of *cdc25*, is essential for the progression of intestinal divisions. Cell Cycle 15: 654–666. 27104746 10.1080/15384101.2016.1146839 PMC4845952

[bib35] L’Hernault, S. W., 2006 Spermatogenesis (February 20, 2006) *WormBook*, ed. The *C. elegans* Research Community WormBook, 10.1895/wormbook.1.85.1, http://www.wormbook.org.

[bib36] Lim S. Kaldis P., 2013 Cdks, cyclins and CKIs: roles beyond cell cycle regulation. Development 140: 3079–3093. 23861057 10.1242/dev.091744

[bib37] Liu K. S. Sternberg P. W., 1995 Sensory regulation of male mating behavior in *Caenorhabditis elegans*. Neuron 14: 79–89. 7826644 10.1016/0896-6273(95)90242-2

[bib38] Liu T. Kim K. Li C. Barr M. M., 2007 FMRFamide-like neuropeptides and mechanosensory touch receptor neurons regulate male sexual turning behavior in *Caenorhabditis elegans*. J. Neurosci. 27: 7174–7182. 17611271 10.1523/JNEUROSCI.1405-07.2007 PMC6794584

[bib39] Loer C. M. Kenyon C. J., 1993 Serotonin-deficient mutants and male mating behavior in the nematode *Caenorhabditis elegans*. J. Neurosci. 13: 5407–5417. 8254383 10.1523/JNEUROSCI.13-12-05407.1993 PMC6576401

[bib40] Maduro M. Pilgrim D., 1995 Identification and cloning of *unc-119*, a gene expressed in the *Caenorhabditis elegans* nervous system. Genetics 141: 977–988. 8582641 10.1093/genetics/141.3.977 PMC1206859

[bib41] Maeda I. Kohara Y. Yamamoto M. Sugimoto A., 2001 Large-scale analysis of gene function in *Caenorhabditis elegans* by high-throughput RNAi. Curr. Biol. 11: 171–176. 11231151 10.1016/s0960-9822(01)00052-5

[bib42] Malumbres M., 2014 Cyclin-dependent kinases. Genome Biol. 15: 122. 25180339 10.1186/gb4184 PMC4097832

[bib43] Mello C. Fire A., 1995 DNA transformation. Methods Cell Biol. 48: 451–482. 8531738

[bib44] Monteiro M. I. Ahlawat S. Kowalski J. R. Malkin E. Koushika S. P., 2012 The kinesin-3 family motor KLP-4 regulates anterograde trafficking of GLR-1 glutamate receptors in the ventral nerve cord of *Caenorhabditis elegans*. Mol. Biol. Cell 23: 3647–3662. 22855524 10.1091/mbc.E12-04-0334 PMC3442412

[bib45] Nass R. Hall D. H. Miller D. M. III Blakely R. D., 2002 Neurotoxin-induced degeneration of dopamine neurons in *Caenorhabditis elegans*. Proc. Natl. Acad. Sci. USA 99: 3264–3269. 11867711 10.1073/pnas.042497999 PMC122507

[bib46] Nelson G. A. Lew K. K. Ward S., 1978 Intersex, a temperature-sensitive mutant of the nematode *Caenorhabditis elegans*. Dev. Biol. 66: 386–409. 700253 10.1016/0012-1606(78)90247-6

[bib47] Ou C. Y. Poon V. Y. Maeder C. I. Watanabe S. Lehrman E. K., 2010 Two cyclin-dependent kinase pathways are essential for polarized trafficking of presynaptic components. Cell 141: 846–858. 20510931 10.1016/j.cell.2010.04.011 PMC3168554

[bib48] Park M. Watanabe S. Poon V. Y. Ou C. Y. Jorgensen E. M., 2011 CYY-1/cyclin Y and CDK-5 differentially regulate synapse elimination and formation for rewiring neural circuits. Neuron 70: 742–757. 21609829 10.1016/j.neuron.2011.04.002 PMC3168547

[bib49] Robertson S. M. Medina J. Lin R., 2014 Uncoupling different characteristics of the *C. elegans* E lineage from differentiation of intestinal markers. PLoS One 9: e106309. 25181289 10.1371/journal.pone.0106309 PMC4152275

[bib50] Russell P. Nurse P., 1987 Negative regulation of mitosis by *wee1*^+^, a gene encoding a protein kinase homolog. Cell 49: 559–567. 3032459 10.1016/0092-8674(87)90458-2

[bib51] Sadhu K. Reed S. I. Richardson H. Russell P., 1990 Human homolog of fission yeast cdc25 mitotic inducer is predominantly expressed in G2. Proc. Natl. Acad. Sci. USA 87: 5139–5143. 2195549 10.1073/pnas.87.13.5139 PMC54277

[bib52] Sawin E. R. Ranganathan R. Horvitz H. R., 2000 *C. elegans* locomotory rate is modulated by the environment through a dopaminergic pathway and by experience through a serotonergic pathway. Neuron 26: 619–631. 10896158 10.1016/s0896-6273(00)81199-x

[bib53] Schedl T. Kimble J., 1988 *fog-2*, a germ-line-specific sex determination gene required for hermaphrodite spermatogenesis in *Caenorhabditis elegans*. Genetics 119: 43–61. 3396865 10.1093/genetics/119.1.43 PMC1203344

[bib54] Segref A. Cabello J. Clucas C. Schnabel R. Johnstone I. L., 2010 Fate specification and tissue-specific cell cycle control of the *Caenorhabditis elegans* intestine. Mol. Biol. Cell 21: 725–738. 20053685 10.1091/mbc.E09-04-0268 PMC2828960

[bib55] Singaravelu G. Chatterjee I. Marcello M. R. Singson A., 2011 Isolation and *in vitro* activation of *Caenorhabditis elegans* sperm. J. Vis. Exp. 47: e2336. 10.3791/2336 PMC3182656 21307834

[bib56] Steimel A. Suh J. Hussainkhel A. Deheshi S. Grants J. M., 2013 The *C. elegans* CDK8 Mediator module regulates axon guidance decisions in the ventral nerve cord and during dorsal axon navigation. Dev. Biol. 377: 385–398. 23458898 10.1016/j.ydbio.2013.02.009

[bib57] Su S. C. Tsai L. H., 2011 Cyclin-dependent kinases in brain development and disease. Annu. Rev. Cell Dev. Biol. 27: 465–491. 21740229 10.1146/annurev-cellbio-092910-154023

[bib58] Sulston J. Dew M. Brenner S., 1975 Dopaminergic neurons in the nematode *Caenorhabditis elegans*. J. Comp. Neurol. 163: 215–226. 240872 10.1002/cne.901630207

[bib59] Sulston J. E. Horvitz H. R., 1977 Post-embryonic cell lineages of the nematode, *Caenorhabditis elegans*. Dev. Biol. 56: 110–156. 838129 10.1016/0012-1606(77)90158-0

[bib60] Sulston J. E. Albertson D. G. Thomson J. N., 1980 The *Caenorhabditis elegans* male: postembryonic development of nongonadal structures. Dev. Biol. 78: 542–576. 7409314 10.1016/0012-1606(80)90352-8

[bib61] Sze J. Y. Victor M. Loer C. Shi Y. Ruvkun G., 2000 Food and metabolic signaling defects in a *Caenorhabditis elegans* serotonin-synthesis mutant. Nature 403: 560–564. 10676966 10.1038/35000609

[bib62] Wang J. Silva M. Haas L. A. Morsci N. S. Nguyen K. C., 2014 *C. elegans* ciliated sensory neurons release extracellular vesicles that function in animal communication. Curr. Biol. 24: 519–525. 24530063 10.1016/j.cub.2014.01.002 PMC4659354

[bib63] Ward S. Hogan E. Nelson G. A., 1983 The initiation of spermiogenesis in the nematode *Caenorhabditis elegans*. Dev. Biol. 98: 70–79. 6345236 10.1016/0012-1606(83)90336-6

[bib64] White J., 1988 The anatomy, pp. 81–122 in *The Nematode Caenorhabditis elegans*, edited by Wood W. B. The Community of *C. elegans* Researchers, Cold Spring Harbor Laboratory Press, New York.

[bib65] Wilson M. A. Hoch R. V. Ashcroft N. R. Kosinski M. E. Golden A., 1999 A *Caenorhabditis elegans wee1* homolog is expressed in a temporally and spatially restricted pattern during embryonic development. Biochim. Biophys. Acta 1445: 99–109. 10209262 10.1016/s0167-4781(99)00027-5

[bib66] Yoon S. Kawasaki I. Shim Y. H., 2012 CDC-25.1 controls the rate of germline mitotic cell cycle by counteracting WEE-1.3 and by positively regulating CDK-1 in *Caenorhabditis elegans*. Cell Cycle 11: 1354–1363. 22421141 10.4161/cc.19755

