# Supplementary material for: cdc-25.4, a Caenorhabditis elegans Ortholog of cdc25, Is Required for Male Mating Behavior
Source: G3 (Bethesda). 2016 Oct 21;6(12):4127–38. doi: 10.1534/g3.116.036129 (PMC5144981; doi:10.1534/g3.116.036129)
Supplement: Supplemental Material [file supp_6_12_4127__index.html]

cdc-25.4, a Caenorhabditis elegans Ortholog of cdc25, Is Required for Male Mating Behavior — Supplemental Material 

# *cdc-25.4*, a *Caenorhabditis elegans* Ortholog of *cdc25*, Is Required for Male Mating Behavior

## Supplemental Material for Shim *et al.*, 2016

**Files in this Data Supplement:**

- Figure S1 - *cdc-25.4(tm4088)* mutant hermaphrodites are fertile. (.pdf, 186 KB)
- Figure S2 - Spermatogenesis and in vitro sperm activation occurred normally in *cdc-25.4(tm4088)* males. (.pdf, 236 KB)
- Figure S3 - Expression pattern of *Pcdc-25.4::gfp* in male tails. Nomarski DIC (A, C, E, G) and fluorescence (B, D, F, H) images of *ExPcdc-25.4::gfp* male tails are shown. (.pdf, 243 KB)
- Figure S4 - Expression pattern of *Pcdc-25.4::gfp* during larval developmental stages. (.pdf, 243 KB)
- Figure S5 - DA and 5-HT treatment could not rescue the defective turning behavior of *cdc-25.4(tm4088)*; *him-5(e1467)* males. (.pdf, 183 KB)
- Figure S6 - Expression pattern of a pan-neuronal marker, *Punc-119::gfp*, was indistinguishable between *him-5(e1467)* and *cdc-25.4(tm4088)*; *him-5(e1467)* adult males. (.pdf, 214 KB)
- Figure S7 - Expression pattern of a pan-neuronal marker, *Punc-119::gfp*, was indistinguishable between *him-5(e1467)* and *cdc-25.4(tm4088)*; *him-5(e1467)* adult males. (.pdf, 214 KB)
- File S1 - Observation of turning behavior in *him-5(e1467)* male. *unc-22(e66)* adult hermaphrodites were used for mating partners. (.zip, 2.45 MB)
- File S2 - *cdc-25.4(tm4088)*; *him-5(e1467)* male failed to turn. *unc-22(e66)* adult hermaphrodites were used for mating partners. (.zip, 1.42 MB)
