# Supplementary material for: cdc-25.4, a Caenorhabditis elegans Ortholog of cdc25, Is Required for Male Mating Behavior
Source: G3 (Bethesda). 2016 Oct 21;6(12):4127–38. doi: 10.1534/g3.116.036129 (PMC5144981; doi:10.1534/g3.116.036129)
Supplement: Supplemental Material [file supp_g3.116.036129_FigureS1.pdf]

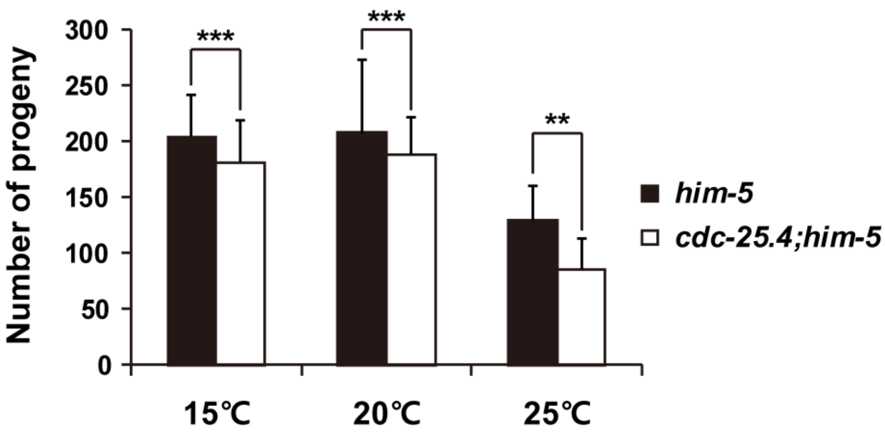

Figure S1 *cdc-25.4(tm4088)* mutant hermaphrodites are fertile. Brood size was examined in *him-5(e1467)* (n=10) and *cdc-25.4(tm4088); him-5(e1467)* (n=10) adult hermaphrodites at three different temperatures (15°C, 20°C, and 25°C). Error bars indicate standard deviation. *P* values were calculated by Student's *t*-test. **$p < 0.05$, ***$p > 0.05$.
