# Supplementary material for: cdc-25.4, a Caenorhabditis elegans Ortholog of cdc25, Is Required for Male Mating Behavior
Source: G3 (Bethesda). 2016 Oct 21;6(12):4127–38. doi: 10.1534/g3.116.036129 (PMC5144981; doi:10.1534/g3.116.036129)
Supplement: Supplemental Material [file supp_g3.116.036129_FigureS2.pdf]

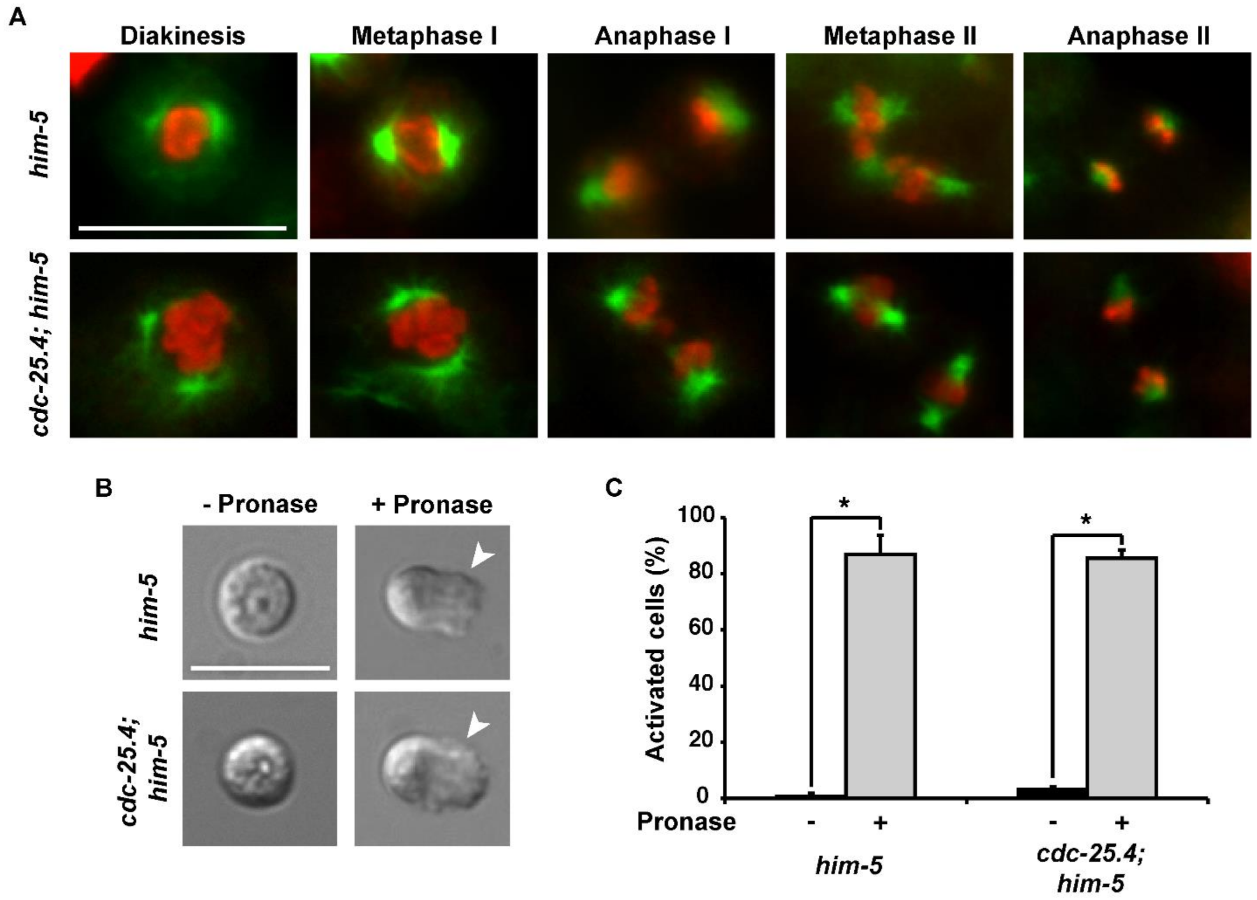

Figure S2 Spermatogenesis and *in vitro* sperm activation occurred normally in *cdc-25.4(tm4088)* males. (A) Meiosis I and II were observed in dissected *him-5(e1467)* (n=31) and *cdc-25.4(tm4088); him-5(e1467)* (n=64) male gonads after co-immunostaining with anti-pH3 (red) and anti-α-tubulin (green). Diakinesis is the final stage of prophase in meiosis I. (B) Sperm were successfully activated *in vitro* after pronase treatment in *cdc-25.4(tm4088); him-5(e1467)* males, as in *him-5(e1467)* control males. Scale bars, 10 μm. (C) Percent activated spermatids in *him-5(e1467)* and *cdc-25.4(tm4088); him-5(e1467)* males (n > 1300). Error bars indicate standard deviation. *P* values were calculated by Student's *t*-test. *$p < 0.001$.
