# Supplementary material for: cdc-25.4, a Caenorhabditis elegans Ortholog of cdc25, Is Required for Male Mating Behavior
Source: G3 (Bethesda). 2016 Oct 21;6(12):4127–38. doi: 10.1534/g3.116.036129 (PMC5144981; doi:10.1534/g3.116.036129)
Supplement: Supplemental Material [file supp_g3.116.036129_FigureS3.pdf]

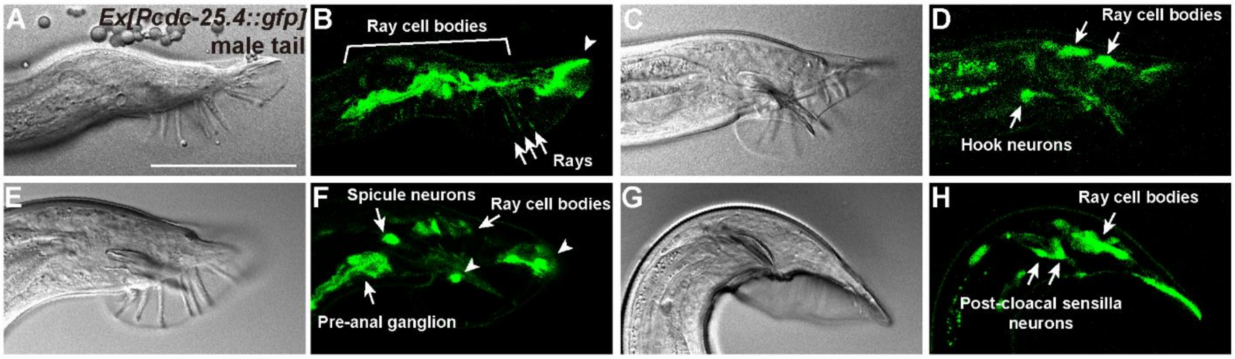

Figure S3 Expression pattern of *Pcdc-25.4::gfp* in male tails. Nomarski DIC (A, C, E, G) and fluorescence (B, D, F, H) images of *Ex[Pcdc-25.4::gfp]* male tails are shown. The *Pcdc-25.4::gfp* transgene was expressed in the ray cell bodies (B, D, F, H), rays (B), hook neurons (HOA or HOB) (D), spicule neurons (SPC, SPD, or SPV) (F), pre-anal ganglion (F) and post-cloacal sensilla neurons (PCA, PCB, or PCC) (H) of male tails. Arrows indicate the loci where *Pcdc-25.4::gfp* was detected. Arrowheads indicate autofluorescence in fan and hook structure. Scale bars, 50 μm.
