# Supplementary material for: cdc-25.4, a Caenorhabditis elegans Ortholog of cdc25, Is Required for Male Mating Behavior
Source: G3 (Bethesda). 2016 Oct 21;6(12):4127–38. doi: 10.1534/g3.116.036129 (PMC5144981; doi:10.1534/g3.116.036129)
Supplement: Supplemental Material [file supp_g3.116.036129_FigureS4.pdf]

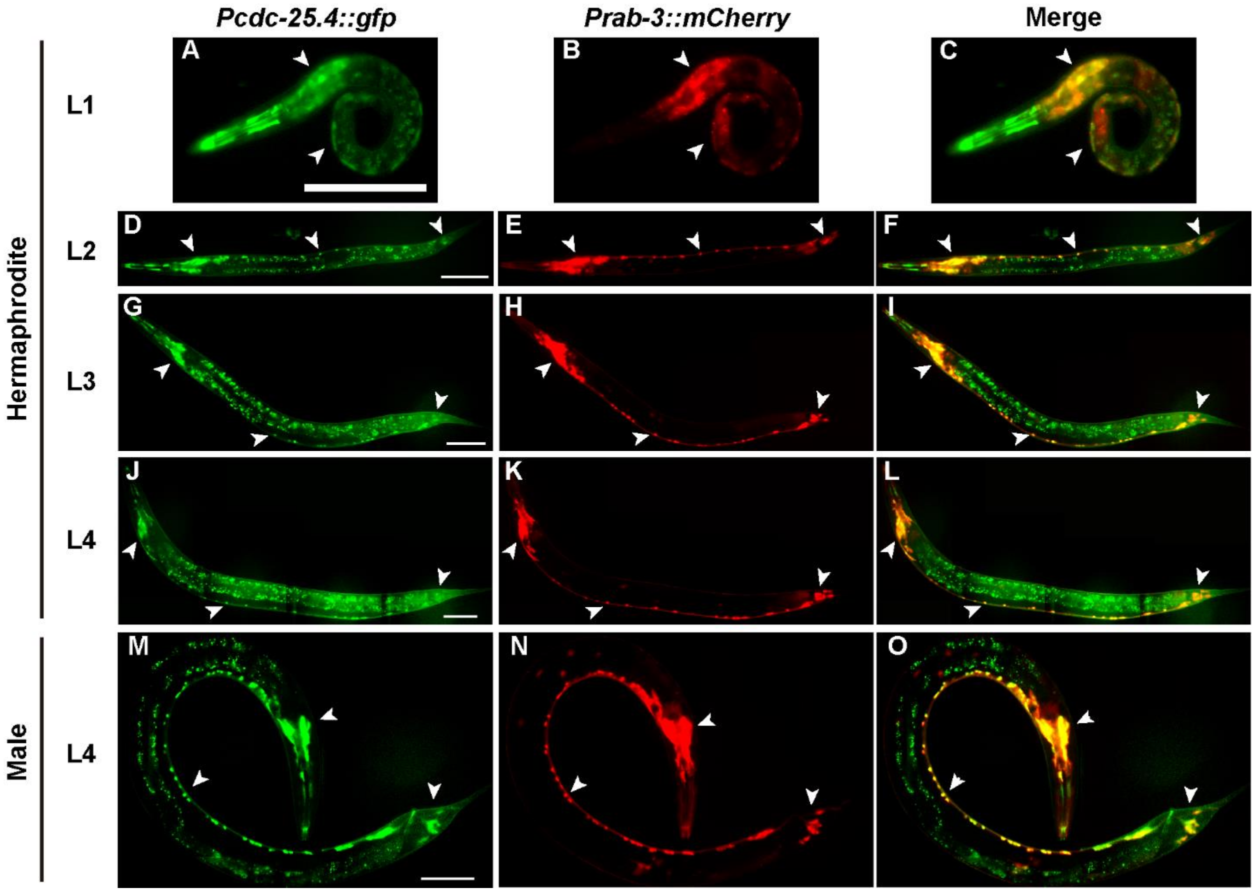

Figure S4 Expression pattern of *Pcdc-25.4::gfp* during larval developmental stages. *Pcdc-25.4::gfp* was expressed in most neurons during larval development in both hermaphrodites and males (A, D, G, J, M). A pan-neuronal marker, *Prab-3::mCherry,* was co-expressed with *Pcdc-25.4::gfp,* and is shown as separate images (B, E, H, K, N) and as merged images (C, F, I, L, O) to illustrate partial overlap between *Pcdc-25.4::gfp* and *Prab-3::mCherry*. Arrowheads indicate the loci where two transgenes were co-expressed. Scale bars, 50 μm.
