# Supplementary material for: cdc-25.4, a Caenorhabditis elegans Ortholog of cdc25, Is Required for Male Mating Behavior
Source: G3 (Bethesda). 2016 Oct 21;6(12):4127–38. doi: 10.1534/g3.116.036129 (PMC5144981; doi:10.1534/g3.116.036129)
Supplement: Supplemental Material [file supp_g3.116.036129_FigureS5.pdf]

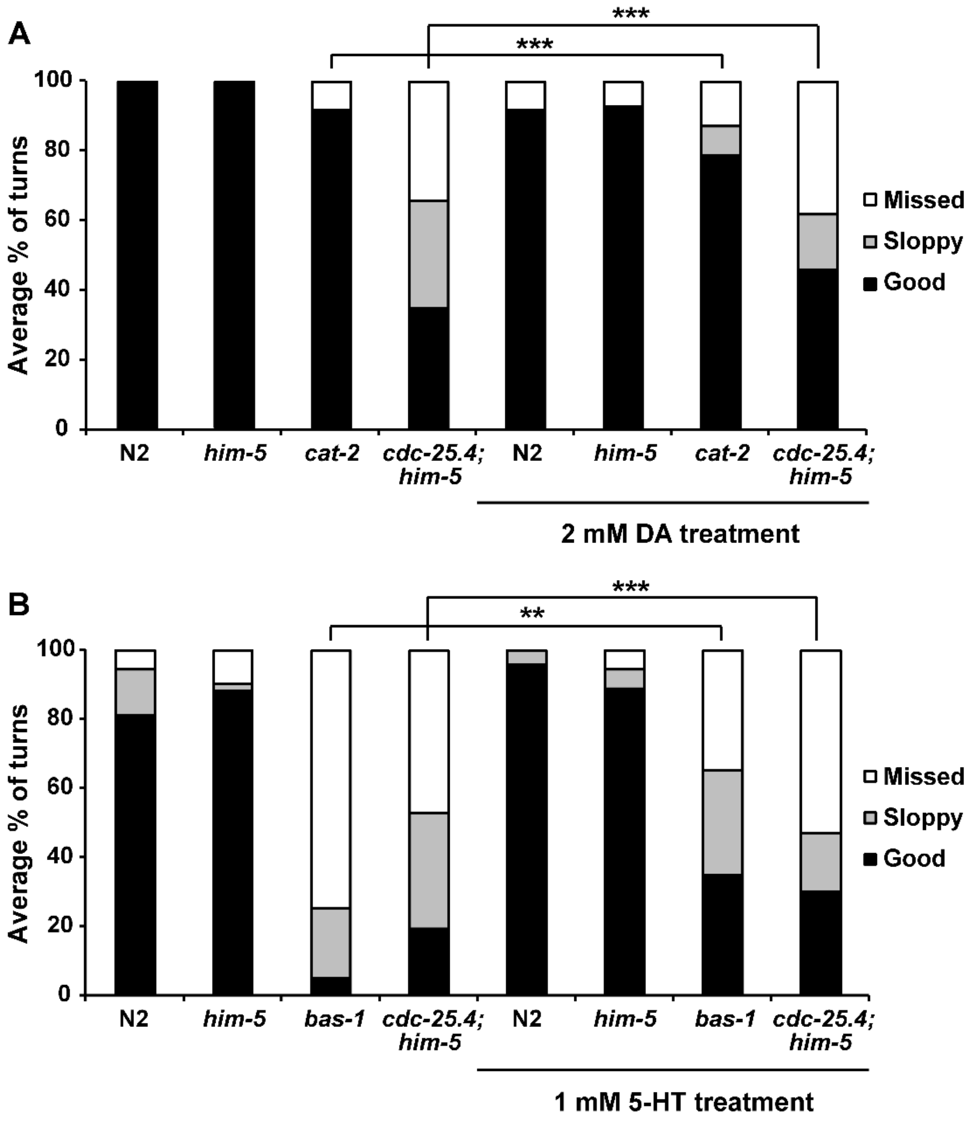

Figure S5 DA and 5-HT treatment could not rescue the defective turning behavior of *cdc-25.4(tm4088); him-5(e1467)* males. (A) Percent distribution of respective turning behavior levels (good, sloppy, or missed) in N2 (n=6), *him-5(e1467)* (n=7), *cat-2(e1112)* (n=7), and *cdc-25.4(tm4088); him-5(e1467)* (n=9) males with or without 2 mM DA treatment. DA treatment to *cdc-25.4; him-5* males could not rescue the defective turning behavior. (B) Percent distribution of respective turning behavior of N2 (n=13), *him-5(e1467)* (n=12), *bas-1(ad446)* (n=12), and *cdc-25.4(tm4088); him-5(e1467)* (n=17) males with or without 1 mM 5-HT treatment. Although 5-HT treatment improved the turning behavior defect of *bas-1(ad446)* serotonin-deficient males, treatment could not rescue the defective turning behavior of *cdc-25.4; him-5* males. *P* values were calculated by Student's *t*-test. **$p < 0.05$, ***$p > 0.05$ including both good and sloppy turns, versus untreated males.
