# Supplementary material for: cdc-25.4, a Caenorhabditis elegans Ortholog of cdc25, Is Required for Male Mating Behavior
Source: G3 (Bethesda). 2016 Oct 21;6(12):4127–38. doi: 10.1534/g3.116.036129 (PMC5144981; doi:10.1534/g3.116.036129)
Supplement: Supplemental Material [file supp_g3.116.036129_FigureS6.pdf]

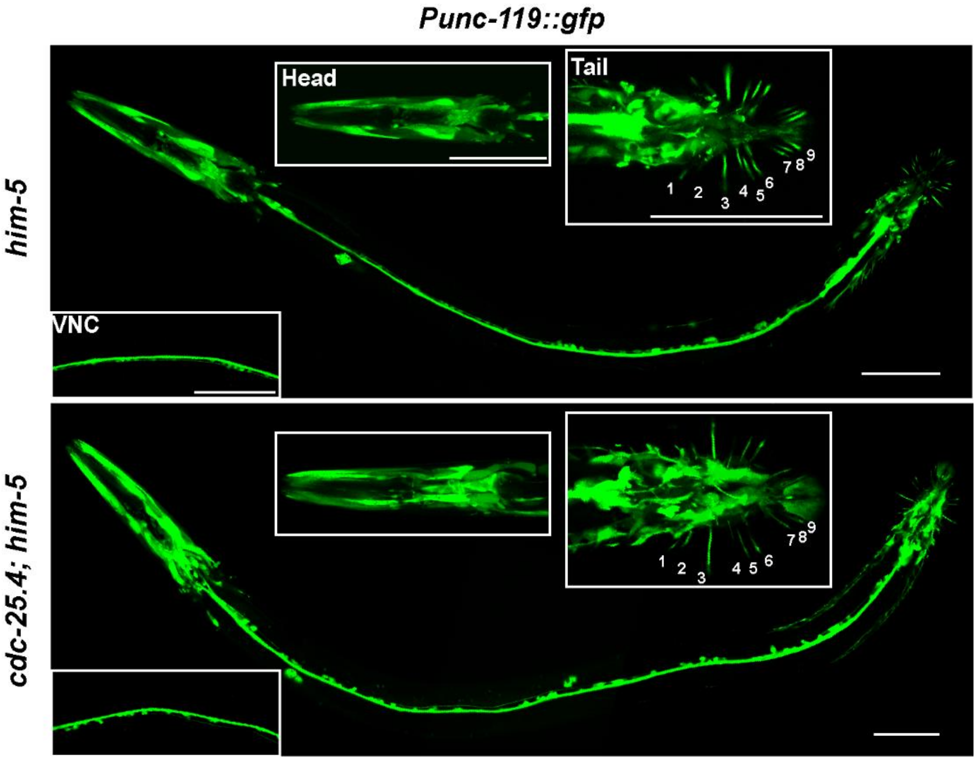

Figure S6 Expression pattern of a pan-neuronal marker, *Punc-119::gfp,* was indistinguishable between *him-5(e1467)* and *cdc-25.4(tm4088); him-5(e1467)* adult males. Insets show *Punc-119::gfp* expression in the head, ventral nerve cord (VNC), and tail. Numbers in the tail images indicate that 9 rays were observed normally in males of both genotypes. Scale bars, 50 μm.
