# Supplementary material for: cdc-25.4, a Caenorhabditis elegans Ortholog of cdc25, Is Required for Male Mating Behavior
Source: G3 (Bethesda). 2016 Oct 21;6(12):4127–38. doi: 10.1534/g3.116.036129 (PMC5144981; doi:10.1534/g3.116.036129)
Supplement: Supplemental Material [file supp_g3.116.036129_FigureS7.pdf]

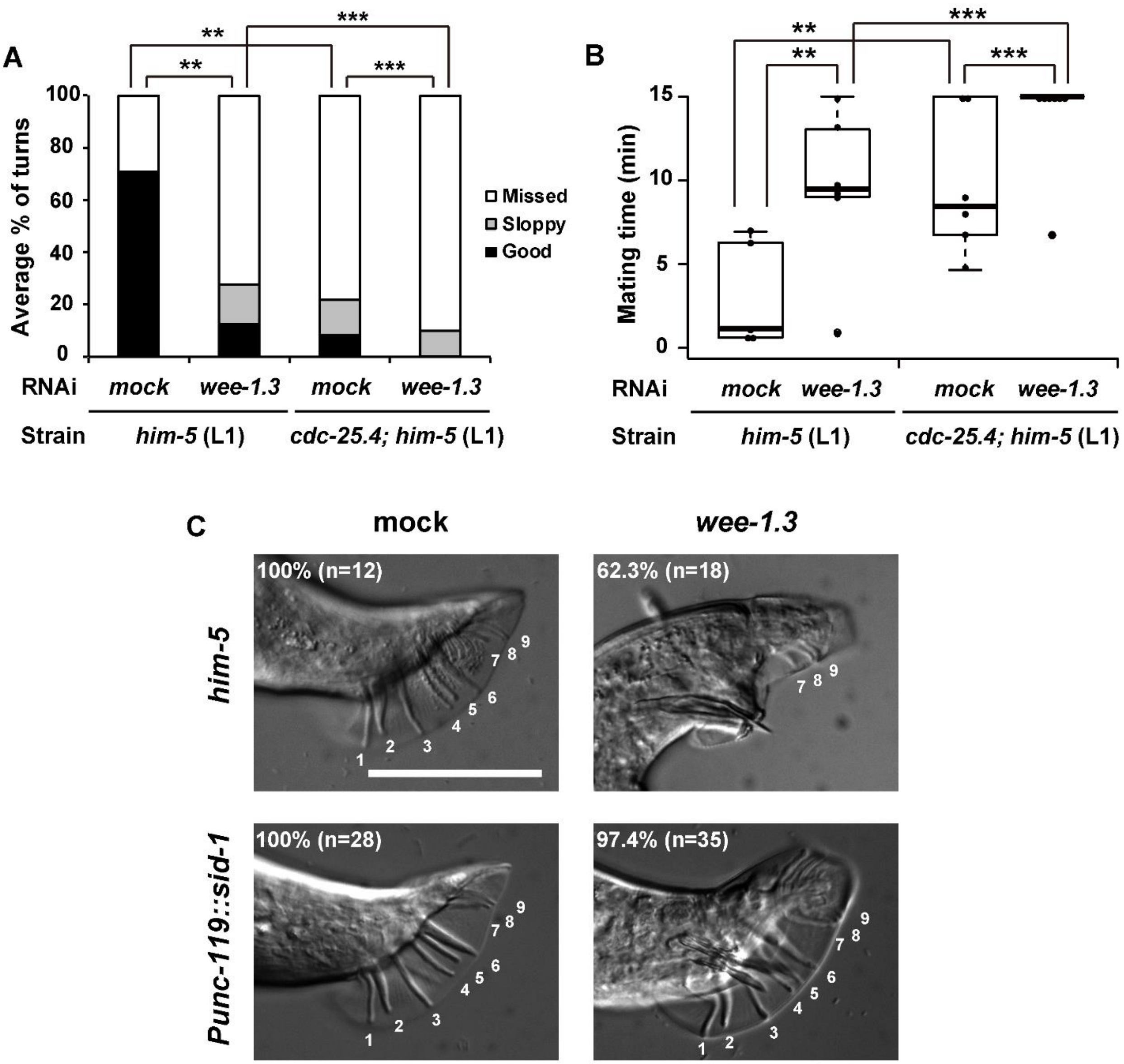


**Figure S7** RNAi depletion of *wee-1.3* did not suppress defective turning behavior of *cdc-25.4(tm4088)* males. (A) Levels of turning behavior in *him-5(e1467)* and *cdc-25.4(tm4088); him-5(e1467)* males, which were classified into good, sloppy, and missed, were measured after *wee-1.3* RNAi depletion at the L1 larval stage. (B) The time period (min) required for mating by *him-5(e1467)* and *cdc-25.4(tm4088); him-5(e1467)* males after *wee-1.3* RNAi depletion at the L1 larval stage. The result is displayed as a box plot. *P* values were calculated by Student's *t*-test. **$p < 0.05$, ***$p > 0.05$. (C) Male tail phenotypes after depletion of *wee-1.3* are shown. 62.3% of *wee-1.3* depleted worms had severe defects in male tail development. Neuron-specific RNAi of *wee-1.3* did not cause abnormality in male tails. Scale bar, 50 μm.
